# Interleukin-38 ameliorates poly(I:C) induced lung inflammation: therapeutic implications in respiratory viral infections

**DOI:** 10.1038/s41419-020-03283-2

**Published:** 2021-01-07

**Authors:** Xun Gao, Paul Kay Sheung Chan, Grace Chung Yan Lui, David Shu Cheong Hui, Ida Miu-Ting Chu, Xiaoyu Sun, Miranda Sin-Man Tsang, Ben Chung Lap Chan, Christopher Wai-Kei Lam, Chun-Kwok Wong

**Affiliations:** 1 Department of Chemical Pathology, The Chinese University of Hong Kong, Hong Kong, China; 2 Department of Microbiology, The Chinese University of Hong Kong, Hong Kong, China; 3 Stanley Ho Centre for Emerging Infectious Diseases, The Chinese University of Hong Kong, Hong Kong, China; 4 Department of Medicine and Therapeutics, The Chinese University of Hong Kong, Hong Kong, China; 5 Institute of Chinese Medicine and State Key Laboratory of Research on Bioactivities and Clinical Applications of Medicinal Plants, The Chinese University of Hong Kong, Hong Kong, China; 6 Faculty of Medicine and State Key Laboratory of Quality Research in Chinese Medicines, Macau University of Science and Technology, Macau, China; 7 Li Dak Sum Yip Yio Chin R & D Centre for Chinese Medicine, The Chinese University of Hong Kong, Hong Kong, China

**Keywords:** Interleukins, Respiratory tract diseases

## Abstract

Interleukin-38 has recently been shown to have anti-inflammatory properties in lung inflammatory diseases. However, the effects of IL-38 in viral pneumonia remains unknown. In the present study, we demonstrate that circulating IL-38 concentrations together with IL-36α increased significantly in influenza and COVID-19 patients, and the level of IL-38 and IL-36α correlated negatively and positively with disease severity and inflammation, respectively. In the co-cultured human respiratory epithelial cells with macrophages to mimic lung microenvironment in vitro, IL-38 was able to alleviate inflammatory responses by inhibiting poly(I:C)-induced overproduction of pro-inflammatory cytokines and chemokines through intracellular STAT1, STAT3, p38 MAPK, ERK1/2, MEK, and NF-κB signaling pathways. Intriguingly, transcriptomic profiling revealed that IL-38 targeted genes were associated with the host innate immune response to virus. We also found that IL-38 counteracts the biological processes induced by IL-36α in the co-culture. Furthermore, the administration of recombinant IL-38 could mitigate poly I:C-induced lung injury, with reduced early accumulation of neutrophils and macrophages in bronchoalveolar lavage fluid, activation of lymphocytes, production of pro-inflammatory cytokines and chemokines and permeability of the alveolar-epithelial barrier. Taken together, our study indicates that IL-38 plays a crucial role in protection from exaggerated pulmonary inflammation during poly(I:C)-induced pneumonia, thereby providing the basis of a novel therapeutic target for respiratory viral infections.

## Introduction

Virus lung infections represent a considerable global health burden, as recognized by WHO’s Battle against Respiratory Viruses initiative^1^. Pneumonia and resultant acute respiratory distress syndrome (ARDS) are the main risk factors in death induced by highly pathogenic respiratory viruses. Most patients who died in the outbreak of severe acute respiratory syndrome (SARS) in 2003, as well as Coronavirus disease 2019 (COVID-19) that is currently pandemic, developed ARDS, septic shock, and/or multiple organ failure^2,3^. Influenza virus-induced pneumonia and the resultant ARDS are also associated with high mortality in critical care patients^4^. In addition, lung inflammation caused by respiratory virus is also the exacerbating factors contributing to chronic airway diseases including asthma and chronic obstructive pulmonary disease (COPD).

Pathological studies showed that imbalanced host immune response to viral pathogenicity contributed to the fatal lung injury in respiratory viral infection, characterized by high viral load, excessive cytokines expression, and overwhelming immune cells influx^5,6^. Therefore, a detailed understanding of the factors that regulate the balance between viral clearance, tissue damage, and resolution of inflammation is necessary for the identification of novel targets for personalized immunotherapy in acute lung inflammation caused by a respiratory virus.

Over the past decades, increasing studies have shed light on the therapeutic potential of anti-inflammatory agents during respiratory viral infection. A combination of anti-viral therapy and anti-inflammatory non-steroidal inhibiting cyclooxygenases (COX) has been shown to improve the survival of mice infected with influenza A/H5N1 virus^6^. Targeting inhibitory pathways such as macrophage CD200R and endothelial S1P1 receptors have been found to reduce immunopathological features in an influenza infection model^7,8^. Moreover, inhaled granulocyte/macrophage colony-stimulating factor was also found to be a promising treatment of pneumonia-associated acute respiratory distress syndrome^9^. Recently, tocilizumab, a monoclonal-antibody for immunosuppressive therapy against IL-6 receptor, has also been explored for COVID-19 therapy^10^. Thus, therapy targeting suppression of pro-inflammatory response is in great urgent to be developed.

Interleukin-38 (IL-38), the novel member of interleukin-1 (IL-1) family, is widely recognized as a pivotal regulator in inflammation. IL-38 shares nearly 41% homology with IL-1 receptor antagonist (IL-1Ra) and 43% homology with IL-36 receptor antagonist (IL-36Ra)^11,12^. IL-38, similar to that of IL-36Ra, functions as the receptor antagonists of IL-36 cytokines including IL-36α, IL-36β, and IL-36γ^13^. IL-38 has been confirmed to be functional in various immune diseases including allergic asthma^14,15^, systemic lupus erythematosus^16^, primary Sjogren’s disease^17^, and rheumatoid arthritis^18^. Unlike IL-36 agonist such as IL-36α that can be increased in murine pulmonary infection, to induce pulmonary inflammation and exacerbate lung injury^19,20^, the potential effects of IL-38 during viral pneumonia remains unknown. It has been reported that upregulated IL-38 can improve the survival in sepsis by targeting suppressive CD4+CD25+ regulatory T (Treg)^21,22^. Importantly, in a lipopolysaccharide (LPS) and cecal ligation and puncture (CLP) induced ARDS model, IL-38 has been proven to be protective by down-regulating Th17 differentiation^23^. Besides, IL-38 protein was found to be overexpressed in the lungs of patients with idiopathic pulmonary fibrosis and lung adenocarcinoma^24,25^. Previously, we have elucidated the anti-inflammatory mechanism of IL-38 in allergic asthma by attenuating T helper (Th)2, Th17, and innate lymphoid type 2 cells (ILC-2) pathway^15^. With regard to the overwhelming inflammatory response in virus pneumonia, these findings implicate that IL-38 might have important effects on host immune response against viral pathogens.

In this study, we investigated here the potential cellular and molecular mechanisms regarding the regulatory properties of IL-38 in the in vitro co-cultured human macrophages with respiratory epithelial cells, upon stimulation with poly(I:C). In addition, we examined the in vivo anti-inflammatory roles of IL-38 in a poly(I:C)-induced murine pneumonia model. Moreover, the extent of systemic IL-38 expression in patients infected with influenza A or B viruses, as well as SARS-CoV-2 was also evaluated to further confirm the regulatory potential of IL-38 in virus pneumonia.

## Patients, Materials and Methods

### Patients and healthy controls

Adults diagnosed with influenza virus A or B viral infections confirmed by PCR and/or immunofluorescence assays at three participating hospital study sites in Hong Kong, and adults diagnosed with SARS-CoV-2 infections by RT-PCR at Princes of Wales hospital in Hong Kong were assessed for study eligibility. Patients with immunosuppressant treatment, pregnancy/lactation, end-stage renal failure, hepatic failure, cardiac failure/arrhythmia, prolonged corrected QT interval >450 msec on electrocardiogram, and allergic history or other medical contraindications to macrolides were excluded. A total of 50 influenza patients, 85 COVID-19 patients, and 59 healthy control participants were enrolled in the study. For influenza patients, peripheral blood samples (10 ml EDTA) were collected on the first day of hospitalization and at convalescence. For COVID-19 patients, plasma samples from each patient were obtained within a few days after onset, with several exceptions sampled at the later time points of hospitalization. Informed written consents were obtained from all recruited subjects. Ethical approval was obtained from Clinical Research Ethics Committee of the Chinese University of Hong Kong-New Territories East Cluster Hospitals. The study was conducted in accordance with the Declaration of Helsinki [ClinicalTrials.gov NCT01779570]. Informed written consents were obtained from all recruited subjects.

### Preparation of Poly(I:C)

High molecular weight polyinosine-polycytidylic acid (Invivogen, San Diego, CA, USA) was prepared according to manufacturer instruction. Briefly, endotoxin-free water provided by the manufacturer was added to poly(I:C) at a final concentration of 1 mg/ml, incubated in a hot water bath (65–70 °C) for 10 min, and allowed to cool down slowly to room temperature to ensure proper annealing. Poly(I:C) solution was then aliquoted and stored at −20 °C until use. Before use, poly(I:C) solution was vortexed to ensure thorough mixing.

### Induction of human monocyte-derived macrophages (HMDMs)

HMDMs were differentiated from human blood mononuclear cells as described^26^. Briefly, fresh human buffy coat was obtained from healthy volunteers of the Hong Kong Red Cross Blood Transfusion Service. Phosphate-buffered saline diluted fresh human blood buffy coat was centrifuged using the 1.082 g/ml isotonic Percoll solution (GE Healthcare Life Sciences) for 20 min at 1800 rpm. After RBC lysis, monocytes were obtained to a purity >93% by magnetic cell sorting (MACS) with anti-CD14 antibody-coated magnetic beads (Miltenyi Biotec, Bergisch Gladbach, Germany) (Fig. S1). CD14+ monocytes were differentiated into macrophages for 7–8 days in RPMI 1640 medium with L-glutamine, 10% fetal bovine serum (FBS), 1% Penicillin-Streptomycin, 1% sodium pyruvate, and 1% glutamax (Gibco Invitrogen Corp, Carlsbad, CA, USA) and 25 ng/ml of granulocyte macrophage-colony stimulating factor at a density of 1.5 × 10^5^/cm^2^.

### Cell culture and reagents

Human bronchial epithelial cell line BEAS-2B cells were purchased from American Type Culture Collections (ATCC, Manassas, Virginia, USA; RRID: CVCL_0168) and maintained in LHC-8 medium (Thermo Fisher Scientific). A549 cell line was purchased from ATCC (RRID: CVCL_0023) and cultured in RPMI 1640 supplemented with 10% FBS. Human BEAS-2B/A549 (1 × 10^5^) cells and HMDMs (3 × 10^5^) were co-cultured with or without rhIL-38 (100 ng/ml) pre-treatment for 30 min, followed by poly(I:C) (20 µg/ml) stimulation for 30 min (for intracellular signaling pathway study), or 20 h (for cytokine study, ICAM-1 determination and RNA-seq). For co-culture, the medium was replaced by RPMI 1640 containing 10% FBS (Thermo Fisher Scientific).

### Flow cytometric analysis of intracellular signaling pathway and ICAM-1

For analyzing the expressions of ICAM-1, cells were stained with mouse anti-human ICAM-1 antibody (Biolegend). The ICAM-1 expressions on A549 cells, BEAS-2B cells, and HMDMs in their monocultures, as well as in the co-cultures of A549/BEAS-2B cells with HMDMs (Co-A549, Co-BEAS-2B, Co-HMDMs) were analyzed using BD FACSVia flow cytometer. For intracellular signaling pathway, co-cultured cells were pre-treated with rhIL-38 (100 ng/ml) for 30 min and stimulated with poly(I:C) (20 µg/ml) for 20 h. Cells were collected and fixed with fixation buffer (Thermo Fisher Scientific) for 30 min at room temperature, followed with intracellular staining permeabilization washing buffer (Thermo Fisher Scientific) at room temperature for 30 min. Cells were then stained with fluorescence dye-conjugated antibodies of mouse anti-human phosphorylated p38, phosphorylated ERK1/2, phosphorylated MEK, phosphorylated STAT1 and STAT3, and phosphorylated IκBα or corresponding isotypic control antibody (BD Pharmingen). After washing, the relative expressions of cell signaling markers in A549 cells, BEAS-2B cells, and HMDMs in the co-cultures of A549/BEAS-2B with HMDMs (Co-A549, Co-BEAS-2B, and Co-HMDMs) were analyzed with BD FACSVia flow cytometer. Mixed cells were distinguished based on CD45 expression.

### Western blot analysis

Cells were washed in ice-cold PBS and lysed in 150 μL RIPA lysis buffer (Thermo Fisher Scientific) supplemented with phosphatase inhibitors (Thermo Fisher Scientific) and Complete Ultra protease inhibitor (Roche Diagnostics). Total protein was measured with the BCA Protein Assay kit (Thermo Fisher Scientific). Equal amount of proteins were loaded to SDS-PAGE and blotted onto PVDF membrane (GE Healthcare Corp, Piscataway, NJ, USA). The membranes were blocked with 3% bovine serum albumin in TBST (0.1% Tween 20) and probed with corresponding primary antibodies including rabbit anti-human β-actin, STAT1, pSTAT1, STAT3, pSTAT3, P38, pP38, MEK, pMEK, ERK1/2, pERK1/2, IκBα, and pIκBα (Cell Signaling Technology (CST), Danvers, MA, USA). HRP-linked anti-rabbit IgG was used as secondary antibodies (CST). Stained protein was visualized using the ECL chemiluminescent detection system (GE Healthcare Corp).

### RNA-sequencing and functional annotations

Total RNA was extracted from co-cultured cells using QIAzol reagent (Qiagen Inc.). mRNA was purified from total RNA (100 ng) using poly-T oligo-attached magnetic beads and fragmented randomly by the addition of fragmentation buffer for library construction. Sequencing libraries were created using NEBNext® UltraTM RNA Library Prep Kit for Illumina® (Illumina Corp, San Diego, CA, USA). Libraries were sequenced on the Illumina HiSeq 4000 platform to generate 150 bp paired-end reads according to the manufacturer’s instructions. The sequenced reads/raw reads containing low quality reads or reads with adaptor were filtered to obtain clean reads, and these clean reads were subsequently mapped to reference genome by HISAT (version 2.0.4). To analyze gene expression level, fragments per kilobase of transcript per million mapped reads were calculated by HTSeq (version 0.6.1). Differential gene expression analysis of eight experiments was performed using the DESeq2 (version 1.10.1, Bioconductor), genes with *P* < 0.05 regarded as differentially expressed. Differentially expressed genes were clustered by hierarchical clustering analysis and further annotated with GO enrichment analysis using ClustrProfiler (v3.8.1).

### Mice

Inbred female C57BL/6 mice (6–8 weeks old) were purchased and housed under specific pathogen-free conditions in the Laboratory Animal Services Center, The Chinese University of Hong Kong (Hong Kong, China). All mice experiments were approved by and followed the guidelines of the Animal Experimentation Ethics Committee of the Chinese University of Hong Kong.

### Establishment of pneumonia murine model induced by poly(I:C)

Female C57BL/6 mice (6–8 weeks old) were anesthetized using inhaled diethyl ether and administered with poly(I:C) (10 mg/kg/mouse) or PBS intranasally (i.n.) per day. Optimized dose of recombinant murine IL-38 (aa 3–152) (800 ng; Adipogen Life Sciences, San Diego, CA, USA) was given by intraperitoneal (i.p.) injection immediately after the poly(I:C) challenge. Mice were then sacrificed at indicated times, and blood, lung, spleen, and BALF were obtained for future analysis.

### Collection of bronchoalveolar lavage fluid (BALF)

BALF was obtained by inserting a 20-gauge catheter into the trachea through which 1 ml of cold PBS was flushed back and forth 3 times. BALF was centrifuged at 1500 rpm for 10 min at 4 °C. Cell-free supernatants were used for the measurement of total protein concentration with a BCA protein assay kit (Thermo Fisher Scientific Inc, Waltham, MA USA). The BALF cell pellet treated with red cell lysis buffer (Beyotime Inc, Jiangsu, China) was re-suspended in PBS for cell count and immuno-phenotyping.

### Histopathological examination

Lungs and trachea were removed from euthanized mice, fixed in 4% paraformaldehyde for paraffin embedding. The whole lung was then sectioned at 5 µm and stained with hematoxylin and eosin (H&E) kits (Beyotime lnc). H&E stained slides were digitized by light microscopy (Leica DM6000B, Leica Microsystems Inc., Buffalo Grove, IL, USA). Quantitative analysis of tissue injury was measured using the standardized lung injury scoring system as described^27^. Lung injury scoring system parameters included the presence of neutrophils in the alveolar space, neutrophils in the interstitial space, hyaline membranes, proteinaceous debris filling the airspace, and alveolar septal thickening.

### Immunohistochemical staining

The lung slides were prepared as described above and incubated in sodium citrate buffer for 20 min at 100 °C. Subsequently, the lung sections were immersed in 3% H_2_O_2_ in PBS for 15 min to block endogenous peroxidase activity, and then incubated with goat serum at room temperature for 10 min to block non-specific binding. For immunostaining, anti-IL-38 monoclonal antibody (1:200; orb339154, Biobryt) was applied at 4 °C overnight. After washing with PBS, the sections were incubated with biotin-conjugated secondary antibody (1:100; SP-9001, ZSGB-BIO) at room temperature for 2 h and visualized with 3′-diaminobenzidine (ZSGB-BIO). The distribution pattern and intensity of IL-38 were captured under light microscopy (Leica DM6000B).

### Measurement of cytokines and chemokines

Human CXCL9, CXCL10, CCL2, CCL5, IL-1β, IL-6, and TNF-α were quantified using Cytometric Bead Array Flex Sets (BD Biosciences, San Jose, CA, USA). Samples were acquired with a BD FACSVia flow cytometer (BD Biosciences) and data was analyzed using BD Biosciences FCAP array software (V3.0). Murine IL-1β, IL-6, IL17A, TNF-α, IFN-γ, CCL2, CCL5, and CXCL10 in serum and lung homogenate were quantified with Mouse Cytokine Milliplex MAP assay kit (Millipore Corporation, Billerica MA, USA) by using Bio-Plex 200 system (Bio-Rad Laboratories, Hercules, CA, USA), or with LEGENDplex™ kits (Biolegend Inc.) by using BD FACSVia flow cytometer. Human IL-36α, IL-36β, IL-36γ, and IL-36Ra were detected using ELISA kits (R&D Systems, Minneapolis, MN, USA). Serum concentrations of murine IL-36α, IL-36β, and IL-36γ were measured using ELISA kits (ELISAGenie Inc, London, UK).

### RNA extraction and QT-PCR

Total RNA from lung tissues, A549 cells, BEAS-2B cells, HMDMs, and co-cultures was extracted using the QIAzol reagent (Qiagen Inc., Valencia, CA, USA), and reverse transcribed into complementary DNA using PrimeScript^TM^ RT Master Mix (Takara Bio Inc., Shiga, Japan). Quantitative real-time PCR reactions were conducted with the SYBR® Primix Ex TaqT (Takara Bio Inc.). GAPDH was used as the internal control and the relative gene expression was calculated by the ΔΔCt quantification method. Quantitect primer assays (Qiagen Inc., Valencia, CA, USA) were used to detect murine IL-1RAPL1. Other primers used for RT-PCR were displayed in Supplemental Table 1.

### Preparation of single-cell suspension and flow cytometric analysis

Lung and spleen tissues were removed from the sacrificed mice. Lungs were cut into small pieces and enzymatically digested with 5 mL RPMI 1640 medium supplemented with 1 mg/ml collagenase (Roche Diagnostics, Rotkreuz, Switzerland) and 30 µg/ml DNase I (Sigma-Aldrich Corp., Saint Louis, MO, USA) for 90 min at 37 °C. Single-cell suspensions of the lung were obtained after filtration through a 70 μm cell strainer. Spleens were mechanically disrupted with 70 μm cell strainer directly to obtain single-cell suspension. Cell pellets were stained with mAb specific for CD45, CD3, CD4, CD8, γδ-TCR, NK1.1, CD11b, Ly6G, F4/80, IL-17A, IL-4, IFN-γ, CD25 and Foxp3 to define myeloid and lymphoid populations. DRAQ7 was used to exclude the dead cells. The cell populations were analyzed with FACSVia flow cytometer (BD Biosciences) and Navios EX Flow Cytometer (Beckman coulter, Inc., Miami, FL, USA).

### Statistics

Each experiment was replicated at least 3 times as independent biological replicates. Unless otherwise noted, all values are expressed as mean ± SEM. Significant comparisons of data between 2 groups were analyzed using 2-tailed Student’s t-test or non-parametric Mann–Whitney U test. Variances of multiple comparisons were analyzed using one-way ANOVA (GraphPad Prism 8.00). Spearman’s correlation coefficient was used to evaluate the correlation between groups. Significance was set at *P < 0.05; **P < 0.01 and ***P < 0.001.

## Results

### IL-38 ameliorates inflammatory responses in co-cultured human respiratory epithelial cells with macrophages upon stimulation by viral poly(I:C)

The primary cells being infected during viral infection are respiratory epithelial cells, and the interaction of respiratory epithelial cells with macrophages of barrier organs constitute the first line of defense against viral pathogens^28^. We therefore co-cultured alveolar epithelial cell line A549 and/or airway epithelial cell line BEAS-2B cells with human monocyte-derived macrophages (HMDMs), to mimic the lung microenvironment in vitro.

**Fig. 1:**
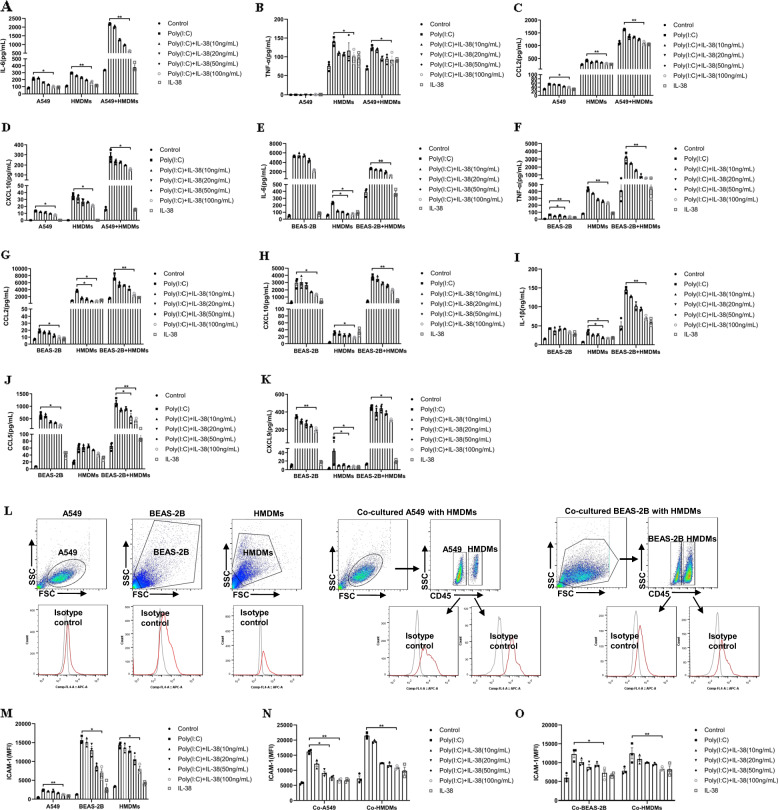
Effects of IL-38 on the cytokines/chemokines released in co-cultured human respiratory epithelial cells with HMDMs. A549 cells (1 × 10^5^) and/or BES-2B cells (1 × 10^5^) with HMDMs (3 × 10^5^) were co-cultured with or without rhIL-38 (100 ng/ml) pretreatment for 30 min, followed by poly(I:C) (20 µg/ml) stimulation for 20 h. (**a**–**k**) Release of cytokines/chemokines in the co-cultured supernatants were measured by Cytometric Bead Array Flex Sets using flow cytometry. (**l**) Gating strategy of flow cytometry for the determination of ICAM-1. (**m**) Flow cytometric analysis of cell surface expression of ICAM-1 on the monoculture of A549 cells, BEAS-2B cells and HMDMs. (**n**, **o**) ICAM expression on A549 cells (Co-A549) in the co-cultured A549 cells with HMDMs, on BEAS-2B cells (Co-BEAS-2B) in the co-cultured BEAS-2B cells with HMDMs, and on HMDMs (Co-HMDMs) in the co-cultured A549/BEAS-2B cells with HMDMs. A549 cells, BEAS-2B cells, and HMDMs in the co-cultures were gated based on CD45 expression. Heat-inactivated (56 °C for 30 min.) human IL-38 (100 ng/ml) was used as the control. Abbreviations: HMDMs, human monocytes derived macrophages; Co-A549, A549 cells in the co-cultured A549 cells with HMDMs; Co-BEAS-2B, BEAS-2B cells in the co-cultured BEAS-2B cells with HMDMs; Co-HMDMs, HMDMs in the co-cultured A549/BEAS-2B cells with HMDMs. All data are shown as the mean ± SEM. Nonparametric Kruskal-Wallis test followed by Dunn’s multiple comparisons test was used to compare results between groups. *P < 0.05 and **P < 0.01.

We investigated whether IL-38 pathways could modulate immunopathology during viral pneumonia in vitro by firstly evaluating the cytokine production in the co-culture thereafter. As shown in Fig. 1, significantly increased expression of IL-6, TNF-α, CXCL10, and CCL2 were observed upon poly(I:C) stimulation in the co-culture of A549 cells with HMDMs compared to the single cultures. IL-6, CCL-2, and CXCL10 were dramatically inhibited by treatment with recombinant human IL-38 protein (rhIL-38) in a dose-dependent manner, and exhibited a significant decrease at IL-38 (100 ng/mL) in A549 cells and HMDMs single cultures and the co-culture (Fig. 1a, c, d). TNF-α was undetectable in the single cultured A549 cells, while it was markedly suppressed by rhIL-38 (100 ng/mL) in the HMDMs and the co-culture (Fig. 1b). Similarly, with rhIL-38 pretreatment, significantly decreased poly(I:C)-induced IL-6, TNF-α, CCL2, and CXCL10 production were observed in the co-culture of BEAS-2B cells with HMDMs (Fig. 1e–h). The release of IL-1β, CCL5, and CXCL9 was also inhibited by rhIL-38 in the co-cultured BEAS-2B cells with HMDMs (Fig. 1i–k). Furthermore, upon stimulation with poly(I:C), cell surface expression of intercellular adhesion molecule (ICAM)-1 was increased in the co-cultured A549 cells with HMDMs compared to the monocultures, and this was suppressed significantly by rhIL-38 (Fig. 1m, n). Similar effects were observed in the co-cultured BEAS-2B cells with HMDMs (Fig. 1m, o). As IL-38 shows high homology to the IL-36 receptor (IL-36R) antagonist (IL-36Ra), IL-38 probably inhibits the activity of the natural ligands of the IL-36R including IL-36α, IL-36β, and IL-36γ. Therefore, the levels of IL-36α, IL-36β and IL-36γ were evaluated in the co-cultures in vitro. We were unable to detect IL-36γ in the co-cultures (data not shown). While we found that poly(I:C) has no effects on the release of IL-36β expressions neither in the co-cultured A549 cells with HMDMs, nor in the co-cultured BEAS-2B cells with HMDMs (Fig. S2), significant increase of IL-36α in the co-cultured A549 cells with HMDMs and in the co-cultured BEAS-2B cells with HMDMs activated by poly (I:C) was observed. To further investigate whether the specific inhibitory mechanism of IL-38 on the biological process is dependent on the IL-36R pathway, we stimulated the co-cultured cells with recombinant IL-36α in the presence of IL-38 and measured the production of TNF-α and IL-6. As shown in Fig. S3, poly (I:C) induced production of TNF-α and IL-6 was reduced with IL-38 treatment in the co-cultured A549 cells with HMDMs as well as in the co-cultured BEAS-2B cells with HMDMs.

Altogether, these results confirmed the direct anti-inflammatory property of IL-38 in vitro by downregulating macrophage-respiratory epithelial cell interaction derived cytokines for innate response, which may be involved in the dysregulated immune response in respiratory viral infections.

### Signaling pathways and target genes related to IL-38 in suppressing poly(I:C)-induced inflammatory mediators in co-culture

**Fig. 2:**
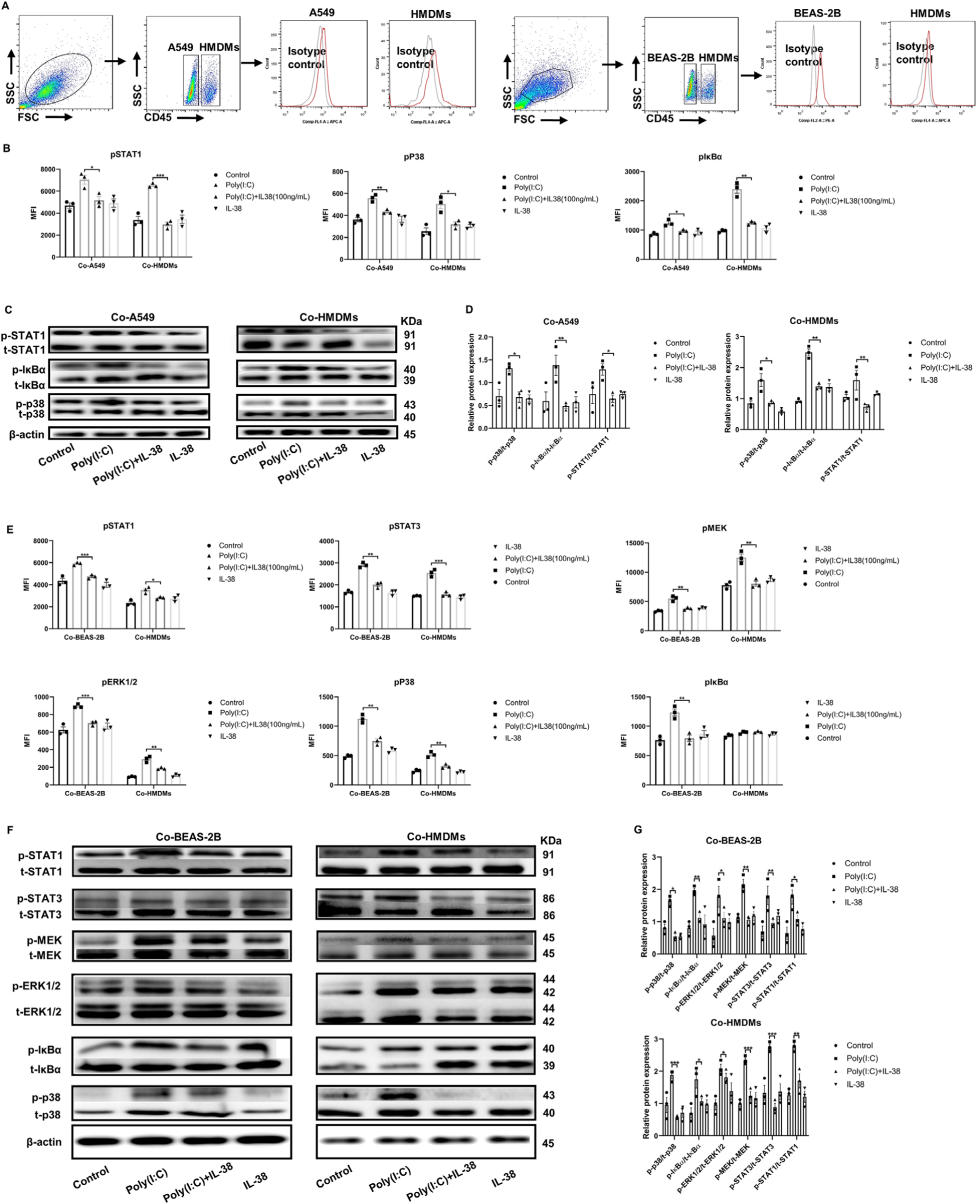

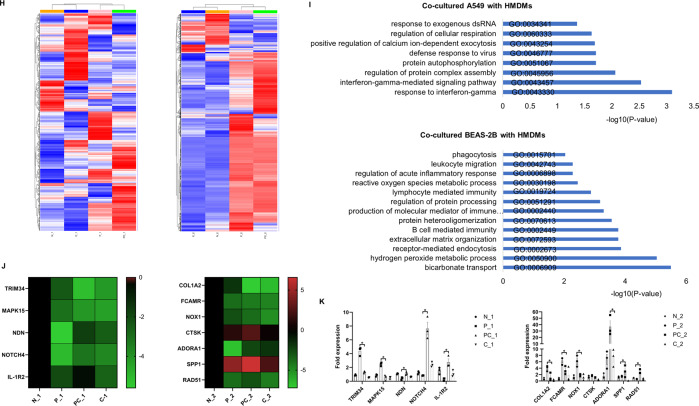
Targeted intracellular signaling pathways and RNA-seq transcriptional profiling identification of IL-38 target genes in the co-cultures. A549 cells (1 × 10^5^) or BEAS-2B cells (1 × 10^5^) with HMDMs (3 × 10^5^) were co-cultured with or without rhIL-38 pretreatment for 30 min, followed by poly(I:C) (20 µg/mL) stimulation for 30 min. Heat-inactivated human IL-38 (100 ng/ml) was used as control. (**a**) Gating strategies for the analysis of signal transducers in the co-cultures. Cells were measured by intracellular staining with specific antibodies, and the absolute MFI values for the phosphorylated signals were used for analysis. A549 cells, BEAS-2B cells, and HMDMs were gated and distinguished based on CD45 expression. (**b**) Flow cytometric analysis of levels of phosphorylated STAT1, p38, and IκBα on A549 cells (Co-A549) and HMDMs (Co-HMDMs) in the co-cultured A549 cells with HMDMs. (**c**) Represented electrophoresis strip of phosphorylated and total STAT1, p38, and IκBα on A549 cells (Co-A549) and HMDMs (Co-HMDMs) in the co-cultured A549 cells with HMDMs detected by Western blot analysis. (**d**) The protein expression of phosphorylated/total STAT1, p38, and IκBα in (**c**). (**e**) Levels of phosphorylated STAT1, STAT3, MEK, ERK1/2, p38, and IκBα in BEAS-2B cells (Co-BEAS-2B) and HMDMs (Co-HMDMs) in the co-cultured BEAS-2B cells with HMDMs detected by flow cytometry. (**f**) Represented electrophoresis strip of phosphorylated and total STAT1, STAT3, p38, ERK1/2, MEK, and IκBα in BEAS-2B cells (Co-BEAS-2B) and HMDMs (Co-HMDMs) in the co-cultured BEAS-2B cells with HMDMs detected by Western blot analysis. (**g**) The protein-expression of phosphorylated / total STAT1, STAT3, p38, ERK1/2, MEK, and IκBα in (**f**). (**h**) Heatmap of the DEGs analyzed by RNA-seq in different groups was shown. (**i**) Analysis showing selected most significantly downregulated pathways determined by GO functional annotations in terms of biological processes. (**j**) The fold change for selected differentially expressed genes based on expression level and relative function in lung inflammation, was displayed in a log_2_-scale heatmap and the corresponding data are shown in Table 2. (K) RT-PCR validation of selected genes indicated in (**j**). Data are shown as the mean ± SEM. Nonparametric Kruskal-Wallis test followed by Dunn’s multiple comparisons test was used to compare results between groups. *P < 0.05, **P < 0.01, and ***P < 0.001. Abbreviations: DEG, differentially expressed gene; PC, poly(I:C) plus IL-38-treated group; P, poly(I:C)-treated group; N, unstimulated group; and C, IL-38-treated group.

To elucidate the in vitro immune mechanisms of the anti-inflammatory activity of IL-38, we investigated the intracellular signaling pathways in the co-culture thereafter using western blot analysis and flow cytometry. As shown in Fig. 2b, upon poly(I:C) stimulation, significantly up-regulated phosphorylation of signal transducers and activators of transcription (STAT)-1, p38 and pIκBa could be observed in the co-cultured A549 cells with HMDMs, and this could be markedly suppressed by exogenous IL-38. We further confirmed this by western blot analysis, which was in consistent with that of flow cytometry (Fig. 2c, d). Similar effects were found in the co-cultured BEAS-2B cells with HMDMs, as decreased poly(I:C)-induced phosphorylation of STAT-1, p38, and pIκBa were also achieved by IL-38 treatment (Fig. 2e–g). In concordance with the more apparent inhibitory effects of IL-38 observed on the cytokine/chemokine release in the co-cultured BEAS-2B cells with HMDMs, apart from STAT-1, p38 and pIκBa, poly(I:C)-induced phosphorylation of STAT-3, ERK1/2 as well as MEK were also significantly inhibited by IL-38 (Fig. 2e–g).

To further identify IL-38 targeted genes in the co-culture, RNA-sequencing (RNA-seq) was used to detect genes with differential expression. Cluster analysis of differentially expressed genes (DEGs) was used to construct a heatmap according to the criteria of *P* < *0.05*. The heatmap showed 131 DEGs between poly(I:C) and poly(I:C) plus IL-38 treated groups in the co-cultured A549 cells with HDMDs, and 184 DEGs in that of co-cultured BEAS-2B cells with HDMDs, respectively (Fig. 2h). Among these DEGs, we identified the most significantly down-regulated genes in the co-culture (Table 1), with fold change summarized in Supplemental Table 2, and performed gene ontology (GO) functional annotations as demonstrated by biological process analysis. Importantly, functional annotations by GO showed that the most significantly down-regulated genes after IL-38 treatment were associated with some important process of host immune response, represented as response to exogenous virus and dsRNA, phagocytosis and lymphocytes mediated immunity (Fig. 2i). We also highlighted several significantly down-regulated genes that have been reported to promote lung fibroblast [collagen type I alpha 2 (*COL1A2*)]^29^, virus replication [Tripartite Motif Containing 34 (*TRIM34*)]^30^, lung inflammation [Mitogen-Activated Protein Kinase 15(*MAPK15*)]^31^ and regulate macrophage function [Fc Fragment of IgA and IgM Receptor (*FCAMR*), NADPH Oxidase 1 (*NOX1*)]^32,33^, and also some significantly up-regulated genes associated with reducing lung fibroblast [Necdin (*NDN*)]^34^, lung inflammation and injury [Notch Receptor 4 (*NOTCH4*), Cathepsin K (*CTSK*), Adenosine A1 Receptor (*ADORA1*), Secreted Phosphoprotein 1 (*SPP1*), Interleukin 1 Receptor Type 2 (*IL-1R2*)]^35–39^, as well as apoptosis and lung injury (*RAD51*)^40^ (Fig. 2j, Table 2). QT-PCR was used to validate the gene expression (Fig. 2k). Based on these data, it is suggested that IL-38 exerts anti-inflammatory properties in vitro.

**Table 1:** The list of genes with most significant change upon IL-38 treatment in the co-cultured respiratory epithelial cells with macrophages.

Cluster significantly up and down-regulated genes	
Co-cultured A549 with HMDMs	Down: *SNCA, CAMK2B, CLTRN, MAPK15, TRIM34, KCNJ8* Up: *IL1R2, CLSTN2, MME, PCDH18, NOTCH4, SLC22A1, CLDN4, MTRNR2L8, PRR4, NDN, CLDN9, CCL15, IGKV2-24, IGKV1-27, IGLV4-69, IGLV2-18, IGHV4-59*
Co-cultured BEAS-2B with HMDMs	Down: *HBB, COL1A2, COL3A1, CA1, HBA2, FCAMR, IL3, SCUBE3, SFRP4, MAOB, NOX1, SPON1, LUM, DCN, SLC4A1, C17orf99, IGKV1-17, IGLV7-43, IGLV3-1, IGHV3-49, IGLV3-9* Up: *CTSK, ADORA1, CHIT1, HAAO, PPBP, SPP1, HIST1H4D, CLEC5A, LPL, GAL, GPR84, RAD51, ATP6V0C, MT1A*

**Table 2:** The fold change for highlighted DEGs in the co-cultured respiratory epithelial cells with macrophages.

Cocultured A549 with HMDMs	Gene_name	N_1	P_1	PC_1	C_1
	TRIM34	0	−2.38	−5.20	−4.20
	MAPK15	0	−3.10	−4.14	−4.38
	NDN	0	−5.61	−1.72	−2.55
	NOTCH4	0	−5.13	−3.20	−3.73
	IL–1R2	0	−2.03	−0.37	−2.69

Cocultured BEAS-2B with HMDMs	Gene_name	N_2	P_2	PC_2	C_2
	COL1A2	0	−1.86	−7.22	−6.63
	FCAMR	0	−3.80	−4.32	−4.64
	NOX1	0	−2.80	−3.84	−5.06
	CTSK	0	1.26	2.70	0.33
	ADORA1	0	−6.86	−2.48	−1.84
	SPP1	0	4.54	6.58	2.36
	RAD51	0	−3.84	−2.88	−4.34

Note: PC, poly(I:C) plus IL-38-treated group; P, poly(I:C)-treated group; N, unstimulated group; and C, IL-38-treated group.

### IL-38 expression in the lung is boosted by poly(I:C) stimulation in vivo to ameliorate poly(I:C)-induced acute lung injury

**Fig. 3:**
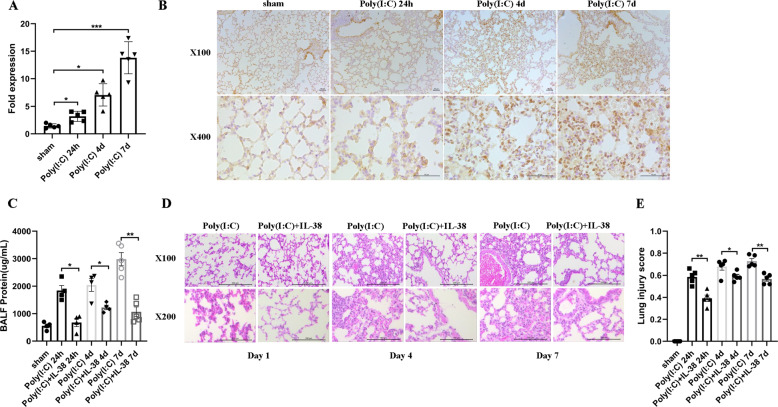
IL-38 expression in the lung is upregulated upon poly(I:C) exposure in vivo and ameliorates poly(I:C)-induced acute lung injury. C57BL/6 mice (*n* = 5) were injected intranasally with poly(I:C) (10 mg/kg/mouse) with/without recombinant murine (rm) IL-38 (800 ng/injection) per day. (**a**) IL-38 mRNA levels in the lung were measured by quantitative real-time PCR (QT-PCR). (**b**) IL-38 protein levels in the lung were detected by immunohistochemistry. (**c**) Total protein concentrations in the BALF were measured with BCA protein assay kit. (**d**) Representative result of hematoxylin and eosin (H&E)-stained lung from mice (*n* = 5 per group) treated with or without rmIL-38 at 24 h, 4 days and 7 days after poly(I:C) injection. (**e**) Histological scores for lung injury assessment in the mice treated with or without rmIL-38 at 24 h, 4 days, and 7 days after poly(I:C) injection. Data are shown as the mean ± SEM of three duplicate tests. Nonparametric Kruskal-Wallis test followed by Dunn’s multiple comparisons test and/or Mann-Whitney test was used to compare the differences between groups. *P < 0.05, **P < 0.01, and ***P < 0.001.

Since in vitro anti-inflammatory roles of IL-38 could account for the potential role of IL-38 in viral pneumonia in vivo, murine model of poly(I:C)-induced pneumonia was established^41–43^. We first examined the production of IL-38 in the lung of mice at 24 h, 4 days and 7 days after poly(I:C) treatment. We detected significantly elevated IL-38 mRNA levels in the lung at 24 h post infection with a higher level increased on day 4 and day 7 (Fig. 3a). Consistent with IL-38 mRNA expression, IL-38 protein in the lung was also elevated at 24 h post infection, with higher levels on day 4 and day 7, as assessed by immunohistochemical staining (Fig. 3b).

We next sought to assess lung injury sequentially after infection. We have preliminarily tested the effects of increasing doses of rmIL-38 (0.5–2 µg) on the poly(I:C)-induced lung injury. As shown in Fig. S4, rmIL-38 treatment decreased bronchoalveolar lavage fluid (BALF) protein concentrations and ameliorated lung injury as evaluated by lung histology. The lowest dose of rmIL-38 (500 ng) decreased BALF total protein slightly (from 1747 to 1416 µg/ml). IL-38 (800 ng and 2 µg) could decrease BALF protein significantly, while there was no significant difference regard to BALF protein between mice treated with 800 ng IL-38 (950 µg/ml) or 2 µg IL-38 (1021 µg/ml) (Fig. S4a), which is in accordance with that of lung injury evaluated by histology (Fig. S4c). Moreover, advanced injection of rmIL-38 (800 ng) at 6 h before poly(I:C) injection could also ameliorate lung injury compared to the mice without IL-38 treatment (Fig. S4b, c). Together, our subsequent experiments applied rmIL-38 (800 ng) immediately after poly(I:C) challenge.

On day 1, 4, and 7, total protein concentrations in BALF of IL-38 treated mice were significantly reduced (Fig. 3c), suggesting downregulated permeability of the alveolar-capillary membrane after IL-38 treatment. Furthermore, exogenous IL-38 administration significantly attenuated poly(I:C)-induced lung injury, with reduced epithelial cell destruction, less inflammatory cells infiltration and edema as reflected by lung histology and corresponding lung injury score (Fig. 3d, e), which was quantified using a standardized lung injury scoring system^27^. The detailed scores for different criteria were summarized in Supplemental Table 3. Importantly, in parallel with what we have observed in the in vitro co-cultures, IL-36α mRNA expression increased most significantly in poly(I:C)-induced lung injury as compared to IL-36β and IL-36γ (Fig. S5a). Furthermore, lung IL-36α protein expressions were significantly induced by poly(I:C) as early as in 24 h hours, and this increase of IL-36α persisted through day 7, indicating IL-36α was the main agonistic ligands that may play roles in poly(I:C)-induced injury (Fig. S5b). Altogether, these data demonstrated that poly(I:C)-induced IL-38 expression is of vital protection in response to acute lung injury.

### Targeting the IL-38 pathways attenuates the immunopathological response induced by poly(I:C)

**Fig. 4:**
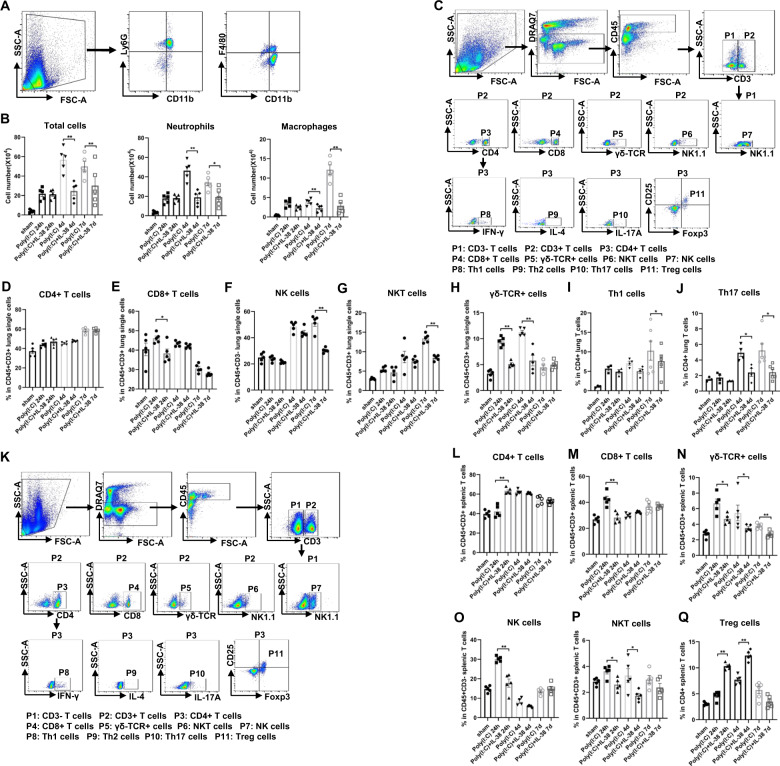
IL-38 reduces leukocyte influx and activation during poly(I:C)-induced lung injury. Bronchoalveolar lavage fluid (BALF), lung, and spleen (*n* = 5 each group) were collected on day 1, 4, and 7 after poly(I:C) injection. (**a**) Gating strategies for the analysis of myeloid cells from BALF cells based on CD11b, F4/80, and Ly6G expressions. (**b**) Number of total cells, neutrophils, and macrophages were counted at indicated times. (**c**, **k**) Gating strategies for the analysis of lymphoid cells from lung and spleen, respectively. Cells were gated based on CD45, CD3, CD4, CD8, γδ-TCR and NK1.1, IFN-γ, IL-4, IL-17A, CD25, and Foxp3 expression for lymphoid populations. DRAQ7 was used to exclude dead cells. (**d**–**j**, **l**–**q**) Percentage of lymphoid cell populations in lung and spleen at indicated time points. Each symbol represents an individual mouse. Data (means ± SEM) are representative of three independent experiments. Mann-Whitney test was used to compare the differences between groups. *p < 0.05, **p < 0.01, and ***p < 0.001.

Given these histological findings, we sought to quantify the immune cell infiltration in the lung. On day 1 post poly(I:C) injection, BALF cell counts are indispensable between IL-38-treated and untreated mice, whereas on day 4 and 7, total amount of leukocytes and corresponding neutrophils and macrophages in BALF reduced strikingly in IL-38-treated group in comparison with groups without IL-38 treatment (Fig. 4b). We also evaluated lung lymphoid cell subsets by characterizing CD4+, CD8+, γδ-TCR+, NK1.1+ T cells, and NK cells, which are involved in the pathophysiology during infection. As early as 24 h after poly(I:C) injection, counts of CD8+ T cells and γδ-TCR+ T cells reduced significantly in IL-38-treated mice, and the reduction of γδ-TCR+ T cells lasted on day 4 (Fig. 4e, h), while significant decrease of NK cells and NKT cells were seen in IL-38-treated mice on day 7, a later time points after poly(I:C) injection (Fig. 4f, g). However, no decrease in CD4+ Th cells was noted between groups with or without IL-38 treatment (Fig. 4d). We next sought to determine the changes in the subtype of CD4+ Th cells by characterizing Th1, Th2, Th17 and Treg cells, which were reported to be the target of IL-38 directly^14,18,40^. Notably, administration of exogenous IL-38 leaded to remarked reduction of Th1 cells on day 7, and Th17 cells on day 4 and 7 (Fig. 4i, j). Similarly, analysis of lymphoid cells in the spleen revealed that the percentage of CD4+ T cells, CD8+ T cells, γδ-TCR + T cells, NK1.1+ T cells, and NK cells were declined markedly after IL-38 administration (Fig. 4l–p). Notably, we observed a strikingly increased percentage of Tregs as early as 24 h in the IL-38 treatment group, as compared to the group without IL-38 treatment (Fig. 4q). These results showed that IL-38 could reduce the recruitment of pro-inflammatory immune cells following poly(I:C) injection during the progress of respiratory viral infection.

### IL-38 decreased cytokine and chemokines productions significantly in poly(I:C) induced lung injury

**Fig. 5:**
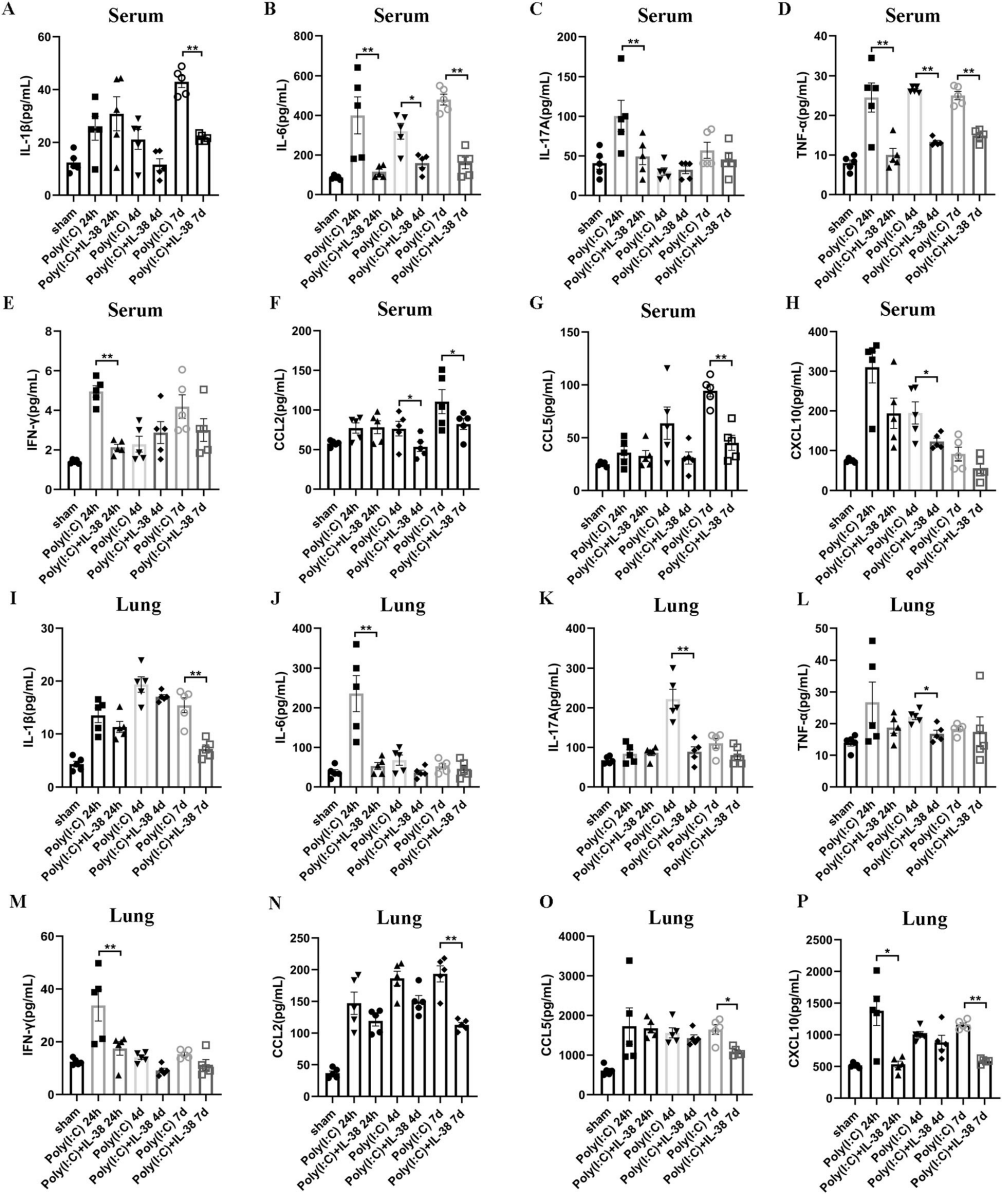
IL-38 reduces cytokine and chemokine production during poly(I:C)-induced lung injury. Serum and lung homogenates (*n* = 3–5 each group) were collected on day 1, 4, and 7 after poly(I:C) injection. Each symbol represents an individual mouse. Specific cytokines and chemokines in the serum (**a**–**h**) and lung (**I–P**) were measured with Mouse Cytokine Milliplex MAP assay kit or LEGENDplex™ kits at indicated times, respectively. Bars represent the mean from at least three different experiments (± SEM). Mann–Whitney test was used to compare the differences between groups. *p < 0.05, **p < 0.01, and ***p < 0.001.

Given that the change of leukocyte recruitment was usually accompanied by cytokine and chemokine alteration during virus-related pneumonia^19,44^, we next sought to analyze the levels of related cytokines and chemokines in the serum and lung homogenates. At 24 h post-infection, serum levels of IL-6, IL-17A, TNF-α, and IFN-γ were greatly reduced in IL-38-treated group (Fig. 5b–e), CCL2 and CXCL10 decreased at day 4 (Fig. 5f, h), IL-1β and CCL-5 decreased on day 7 in the IL-38-treated group compared with group without IL-38 treatment (Fig. 5a, g). Along with this observation, we also observed significantly lower levels of IL-6, IFN-γ, and CXCL-10 24 h post-infection (Fig. 5j, m, p), IL-17A and TNF-α 4 days post-infection (Fig. 5k, l), and IL-1β, CCL2 and CCL5 7 days post-infection in the lung of IL-38-treated mice (Fig. 5i, n, o), compared with mice without IL-38 administration. In the context of lower inflammation, these in vivo results suggest that the protection effects of IL-38 in the poly(I:C)-induced lung injury may be explained by the ameliorated immunopathology.

### Clinical relevance of IL-38 in patients with respiratory viral infections

**Fig. 6:**
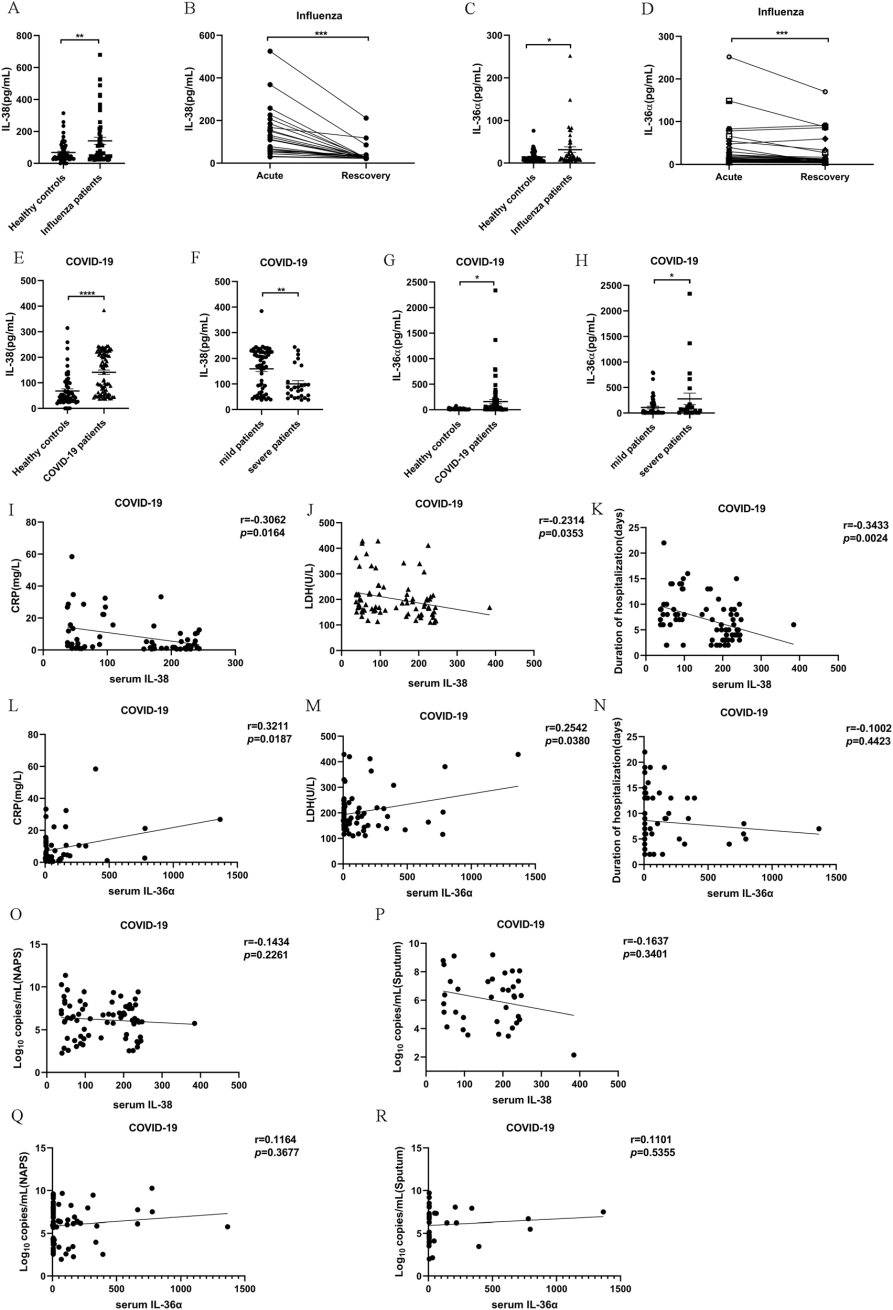
Circulating IL-38 concentration was increased in patients with influenza and SARS-CoV-2 infection. (**a**, **e**) Serum IL-38 concentration was measured by using ELISA of healthy controls (*n* = 59), influenza patients (*n* = 50), and COVID-19 patients (*n* = 85) on the day of hospitalization. Each dot represents a measurement of an individual patient, with horizontal lines denoting medians. (**b**) Serum IL-38 concentration of influenza patients (*n* = 24) in the acute and recovery phases was measured by ELISA. (**c**, **g**) Serum IL-36α concentration was measured by using ELISA in healthy controls (*n* = 50), influenza patients (*n* = 44), and COVID-19 (*n* = 79) patients on the day of hospitalization. (**d**) Serum IL-36α concentration of influenza patients (*n* = 45) in the acute and recovery phases was measured by ELISA. (**f**) Serum IL-38 concentrations in COVID-19 patients with mild (*n* = 59) and severe (*n* = 26) symptoms. (**h**) Serum IL-36α concentrations in COVID-19 patients with mild (*n* = 55) and severe (*n* = 24) symptoms. (**i**–**k**) Correlation analysis of serum IL-38 concentration with CRP levels, LDH levels, duration of hospitalization in patients with SARS-CoV-2 (*n* = 61, 82, 76, respectively). (**l**–**n**) Correlation analysis of serum IL-36α concentration with CRP levels, LDH levels, duration of hospitalization in patients with SARS-CoV-2 (*n* = 53, 67, 61, respectively). (**o**–**p**) Correlation analysis of serum IL-38 levels with viral load in nasopharyngeal swab specimens (**o**) and sputum (**p**) of patients with SARS-CoV-2 (*n* = 73, 36, respectively). (**q**, **r**) Correlation analysis of serum IL-36α levels with viral load in nasopharyngeal swab specimens (**q**) and sputum (**r**) of patients with SARS-CoV-2 (*n* = 62, 34, respectively). Data were shown as (means ± SEM). Student paired *t* test and/or Mann–Whitney test was used to compare the differences between groups. Spearman correlation coefficient was used in the statistics for correlation analysis. *P < 0.05, **P < 0.01, ***P < 0.001, and ****P < 0.0001.

Giving the protective role of IL-38 in poly(I:C) induced lung injury both in vivo and in vitro, and aimed at clarifying the controversial expression and effects of IL-38 in respiratory viral infections, we further evaluated the relevance of IL-38, IL-36 including IL-36α, IL-36β, IL-36γ, together with IL-36Ra in clinical patients with respiratory viral infections. We first measured the circulating IL-38, IL-36α, IL-36β, IL-36γ, and IL-36Ra concentration in patients infected with influenza, a very common type of respiratory viral infections. There were 50 adults with influenza A or B infection enrolled in the study. The basic demographics and clinical characteristics of the patients are summarized in supplemental Table 4. As shown in Fig. 6a, serum IL-38 concentrations were significantly elevated in influenza patients on the day of hospital admission, compared with health individuals. A significant increase of circulating IL-36α was also observed in influenza patients when compared to healthy controls (Fig. 6c). In addition, there was a significantly decreased serum IL-38 as well as IL-36α concentration in influenza patients after they had recovered from acute infection (Fig. 6b, d), these results drive us to hypothesize that circulating IL-38 might associate with the process of respiratory viral infection. To further confirm this, we also validate the clinical association of IL-38 and IL-36α in patients infected with SARS-CoV-2, another type of respiratory viral infection that is pandemic worldwide at present. Totally, 85 COVID-19 patients were enrolled in the study, and demographics and clinical characteristics of patients were presented in supplemental Table 5. Similarly, we found strikingly increased serum IL-38 and IL-36α concentration in COVID-19 patients (Fig. 6e, g). COVID-19 patients were further divided into two different groups based on clinical severity (supplemental Table 5). Importantly, severe COVID-19 patients displayed significantly lower IL-38 concentrations, and higher IL-36α levels compared with non-severe patients (mild patients) (Fig. 6f, h). Moreover, we observed that circulating IL-38 concentrations showed a significantly negative correlation with serum C-reactive protein (CRP) (Fig. 6i), lactate dehydrogenase (LDH) (Fig. 6j), and duration of hospitalization (Fig. 6k) of COVID-19 patients, whereas IL-36α levels were positively correlated with serum CRP (Fig. 6l) and LDH (Fig. 6m), with no significant correlation regarding the duration of hospitalization (Fig. 6n). Although with the decreasing of IL-38 and the increasing of IL-36α levels, there was the tendency of increasing viral loads in nasopharyngeal swab specimens (NAPS) as well as in sputum, there were no significant correlations between serum IL-38 or IL-36α concentrations with viral load (Fig. 6o–r). In addition, no significant difference was observed regarding the circulating IL-36β and IL-36γ expressions both in influenza and COVID-19 patients (Fig. S6a, b). Different from IL-38 and IL-36, IL-36Ra was unable to be detected in influenza patients and COVID-19 patients (data not shown). Overall, these findings further demonstrated elevated anti-inflammatory IL-38 expression in response to respiratory viral infection, and the clinical role of IL-38 in disease severity and inflammation, suggesting IL-38 as a potential therapeutic target for respiratory viral infections.

## Discussion

Dysfunctional immune responses are often associated with fatal outcomes during respiratory viral infection, and downregulation of excessive immune response is crucial in minimizing the severe immunopathology. Many studies targeting pro-inflammatory molecules are being developed in an attempt to mitigate viral-related lung injury. We herein report that IL-38, a novel anti-inflammatory cytokine with suppressive properties, exerts immunomodulatory roles in respiratory viral infections.

Upon respiratory infection, the pathogenic virus principally targets airway epithelial cells, alveolar epithelial cells, vascular endothelial cells, and immune cells including macrophages to subsequently trigger local immune responses^28,45^, the abnormal function which is the main cause of host dysregulated immune response. The present in vitro result showed that IL-38 exhibited profound anti-inflammatory properties in the co-culture of respiratory epithelial cells with macrophages in a dose-dependent manner by inhibiting the production of main cytokines and chemokines, especially IL-6, TNF-α, and viral infection-related Th1 chemokine CXCL10 through STAT1, STAT3, and MAPK pathways including ERK1/2 and p38, MEK and NF-κB signaling pathways, the over-production of which have been shown to associate with more severe clinical illness during viral infection^46^. The elevated release of IL-36α in the co-cultures and IL-38 displayed anti-inflammatory effects on IL-36α induced pro-inflammatory cytokine release in the co-culture indicate that the anti-inflammatory activity of IL-38 may be associated with its counteracting effect on IL-36α-induced biological process in the co-culture, at least partly.

**Fig. 7:**
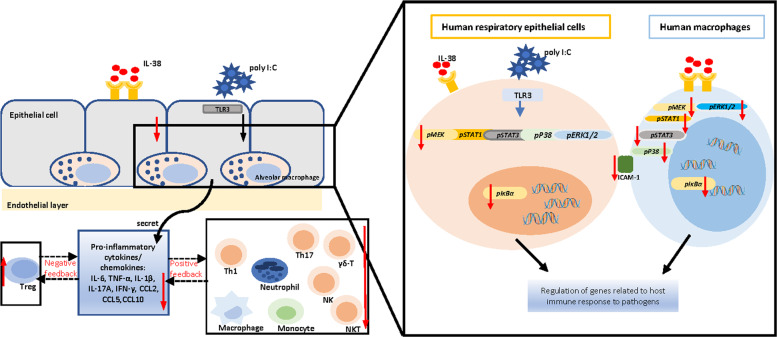
The role and molecular mechanism of IL-38 in respiratory viral infection. IL-38 was able to alleviate inflammatory responses in vitro by inhibiting poly(I:C)-induced over production of pro-inflammatory cytokines and chemokines through intracellular STAT1, STAT3, p38 MAPK, ERK1/2, MEK and NF-κB signaling pathways. IL-38 targeted genes were associated with host innate immune response to virus in vitro. In animal study, IL-38 could mitigate poly(I:C)-induced lung injury by suppressing inflammatory responses by upregulating Treg cells but downregulating Th1, Th17, NK, NKT and γδ T cells.

Correspondingly, our results of RNA-seq transcriptomic profiling to delineate the target genes of IL-38 in the co-culture intriguingly uncovered that the most significantly downregulated genes with IL-38 treatment are associated with host inflammatory response. Notably, we highlighted several target genes that have been linked with lung injury. For example, deficiency of CTSK can aggravate lung injury in mice exposed to hyperoxia^36^. It has been reported that SPP1 is a determinant during lung development and lack of SPP1 may cause deteriorated lung function^38^. Taken together, we explored in vitro that the modulation of inflammation is the mechanism by which IL-38 mitigates the lung injury (Fig. 7).

In further investigation, we established the viral-related TLR3 ligand poly(I:C)-induced mice model of pneumonia, which is widely used for the experimental study of respiratory viral infection. Consistent with our in vitro study, elevated IL-38 levels of the lung tissue in response to poly(I:C)-mediated pulmonary inflammation further implies the regulatory potential of IL-38 in mice with viral-related pneumonia. IL-38 was previously reported to be mainly expressed in the basal epithelia of skin, proliferating B cells of the tonsils, and in spleen, heart, as well as lung^24,47^. Therefore, further studies are required to explore the main sources of elevated IL-38 expression upon poly(I:C) stimulation. We found that lung injury could be mitigated in mice treated with IL-38, as indicated by less BALF protein, reduced lung injury score, and decreased early accumulation of macrophages and neutrophils. These results are in concordance to the previous findings demonstrating that the exuberant recruitment of neutrophils contributes to lung endothelial injury and promotes the progression of acute lung injury^48^, and excessive monocytes influx into alveolar space contributed to lung epithelial cell damage through TRAIL-mediated apoptosis during influenza^49^. With IL-38 treatment, we also found that CD8+ cytotoxic T (Tc) cells decreased. Correspondingly, CD8+ Tc cells were proved to be related to lung damage in response to respiratory viral infection^50,51^. Intriguingly, we found that IL-38 upregulated splenic CD4+ CD25+ Foxp3+ Tregs, while Treg deficiency would lead to severe acute lung damage and decreased blood oxygen concentration during respiratory viral infection^52^. Previously, we have reported that during allergic asthma, IL-38 exhibited regulatory roles on Tregs^15^, and IL-38 has also been proved to have protective effects in sepsis by targeting Tregs^21^. However, it still requires further evidence regarding the specific regulation of IL-38 on Treg cells during respiratory viral infection.

In our study, cytokine analysis revealed that IL-38-mediated the reduction of myeloid and lymphoid cells was accompanied by the release of a panel of pro-inflammatory cytokines and chemokines including IL-17, CXCL-1, CXCL-10, CCL-2, and CCL-5, which have been shown to be elevated and correlated with disease severity in virus-related pneumonia^53^. During respiratory viral infection, the cellular source of IL-17 in the lungs was found to be γδ-T cells^54^. Correspondingly, as we observed decreased γδ-T cells in IL-38-treated mice at the early stage after poly(I:C) injection, the decreased IL-17 production could partly be explained by the reduction of γδ-T cells. We found ameliorated lung inflammation by IL-38 was also associated with decreased systemic production of IL-6, TNF-α, and IL-1β, three pro-inflammatory cytokines that are generally elevated to promote the regression of respiratory viral infection during cytokine storm^55^. IL-38 mediated decrease of neutrophils may partly explain for this reduction, as neutrophils are the potential cellular sources of TNF-α and IL-1β^56,57^. In line with our in vitro results, the increase of lung IL-36α expression remained through day 7, thereby indicating that IL-36α is the main IL-36 agonist that may play roles in poly(I:C)-induced lung injury, and that the protective role of IL-38 in poly(I:C)-induced lung injury may be associated with its antagonism on IL-36α-induced pro-inflammatory cytokines induction. Taken together, our in vivo murine study has further provided evidence that IL-38 mediated immunoregulatory mechanism is capable of relieving the immunopathology and is of vital protection during respiratory viral infection (Fig. 7).

Finally, we demonstrated for the first time the clinical relevance of IL-38 expression in patients with influenza virus and SARS-CoV-2 infection, resulting in three main observations: 1) circulating IL-38 concentration together with IL-36α was significantly elevated in patients with respiratory viral infections; 2) there was a significant decrease of IL-38 as well as IL-36α concentration after recovery from the acute infection, suggesting the predictive role of IL-38 in the prognosis of respiratory viral infection; and 3) severe COVID-19 patients displayed lower IL-38 and higher IL-36α concentration than non-severe patients, and circulating IL-38 levels showed a negative correlation with circulating inflammation-related CRP and LDH levels, as well as duration days of hospitalization, while levels of IL-36α correlated positively with disease severity, which further support our in vivo and in vitro findings that effects of IL-38 during virus pneumonia may be associated with IL-38 counteracting the pro-inflammatory effects of IL-36α. Our results, therefore, demonstrate the regulatory and protective potential of IL-38 in response to respiratory viral infection and providing important basis for future translational studies.

## Conclusion

We have demonstrated for the first time that IL-38 expression was extendedly upregulated in response to different types of respiratory virus infection, in an attempt to suppress the inflammation. By using poly(I:C) as the mimic of common virus infection, we have revealed the involvement of regulatory IL-38 by modulating detrimental host responses during poly(I:C)-induced lung injury. Therefore, our findings may provide insight into the development of therapeutic agents to ablate the inflammatory pathology that leads to severe acute lung injury.

## Supplementary information

Supplemental Figure Legends

Figure S1

Figure S2

Figure S3

Figure S4

Figure S5

Figure S6

Supplemental Table 1

Supplemental Table 2

Supplemental Table 3

Supplemental Table 4

Supplemental Table 5
