# Supplementary material for: Interleukin-38 ameliorates poly(I:C) induced lung inflammation: therapeutic implications in respiratory viral infections
Source: Cell Death Dis. 2021 Jan 7;12(1):53. doi: 10.1038/s41419-020-03283-2 (PMC7790341; doi:10.1038/s41419-020-03283-2)
Supplement: Supplementary file 1 — Supplemental Figure Legends [file 41419_2020_3283_MOESM1_ESM.docx]

**Supplementary** **Figure Legends**

**Figure S1.** Induction and purity of HMDMs. Human peripheral CD14+ monocytes sorted by CD14 magnetic beads was cultured with GM-CSF (25 ng/ml) for 7 consecutive days. (A) Flow cytometric analysis of CD14+ cells in the PBMC before separation and purity of sorted CD14+ monocytes by magnetic cell sorting with anti-CD14 antibody-coated magnetic beads. (B) Representative morphological sections of HMDMs on the first day (day 1), 4 and 7 days after GM-CSF stimulation (day 4 and 7) under light microscopy.

**Figure S2.** Induction of IL-36α and IL-36β in the *in vitro* co-cultures activated by poly(I:C). A549 cells (1x10^5^) or BES-2B cells (1x10^5^) with HMDMs (3x10^5^) were co-cultured with or without rhIL-38 (100 ng/ml) pretreatment for 30 min, followed by poly(I:C) (20 µg/ml) stimulation for 20 hours. (A-D) Release of IL-36α and IL-36β in the co-cultured supernatants were measured by ELISA. Mann-Whitney test was used to compare the differences between groups. **P* < 0.05, ***P* < 0.01.

**Figure S3.** IL-38 inhibits IL-36α-induced cytokine release in the co-cultured respiratory epithelial cells with HMDMs. A549 cells (1x10^5^) or BEAS-2B (1x10^5^) cells with HMDMs (3x10^5^) were co-cultured with or without rhIL-38 pretreatment for 30 min, followed by IL-36α (100 ng/mL) stimulation for 20 h. Release of IL-6 and TNF-α in the supernatants from co-cultured A549 cells with HMDMs (A-B), and co-cultured BEAS-2B cells with HMDMs (C-D) were measured by ELISA. Data are shown as the mean ± SEM. Mann-Whitney test was used to compare the difference between groups. **p* < 0.05.

**Figure S4.** Dose response and preventive effects of IL-38 on poly(I:C)-induced acute lung injury. C57BL/6 mice (n=4) was injected intranasally with poly(I:C) (10 mg/kg/mouse) and sacrificed 24h thereafter. Recombinant murine (rm) IL-38 (500 ng, 800 ng, 2 µg /injection) was injected intraperitoneally immediately after (A) or 6h before poly(I:C) injection (B), PBS was used in the same fashion as control. Total protein concentration in the BALF were measured with BCA protein assay kit. (C) Representative examples of hematoxylin and eosin (H&E)-stained lung. Data are shown as the mean ± SEM of three duplicate tests. Nonparametric Kruskal-Wallis test followed by Dunn’s multiple comparisons test and/or Mann-Whitney test was used to compare the differences between groups. **P* < 0.05.

**Figure S5.** Expression of IL-36α, IL-36β and IL-36γ in poly(I:C)-induced lung injury**.** C57BL/6 mice (n=4-6) were intranasally injected with poly(I:C) (10 mg/kg/mouse) or PBS, and lungs were collected at indicated times post poly(I:C) injection. (A) mRNA expressions of IL-36α, IL-36β and IL-36γ in the lung at 24h after poly(I:C) injection were measured by QT-PCR. (B) Protein expressions of IL-36α in the lung homogenates were measured by ELISA. Data are shown as the mean ± SEM. Mann-Whitney test was used to compare the difference between groups. **P* < 0.05, ***P* < 0.01.

**Figure S6.** Circulating IL-36γ and IL-36β levels in patients with influenza and SARS-CoV-2 infection. (A-B) Serum IL-36γ and IL-36β concentrations were measured by using ELISA in healthy controls (n=50), influenza patients (n=44) and COVID-19 patients (n=79) on the day of hospitalization. Each dot represents a measurement of an individual patient, with horizontal lines denoting medians.
