# Supplementary figures and images for: Interleukin-38 ameliorates poly(I:C) induced lung inflammation: therapeutic implications in respiratory viral infections
Source: Cell Death Dis. 2021 Jan 7;12(1):53. doi: 10.1038/s41419-020-03283-2 (PMC7790341; doi:10.1038/s41419-020-03283-2)

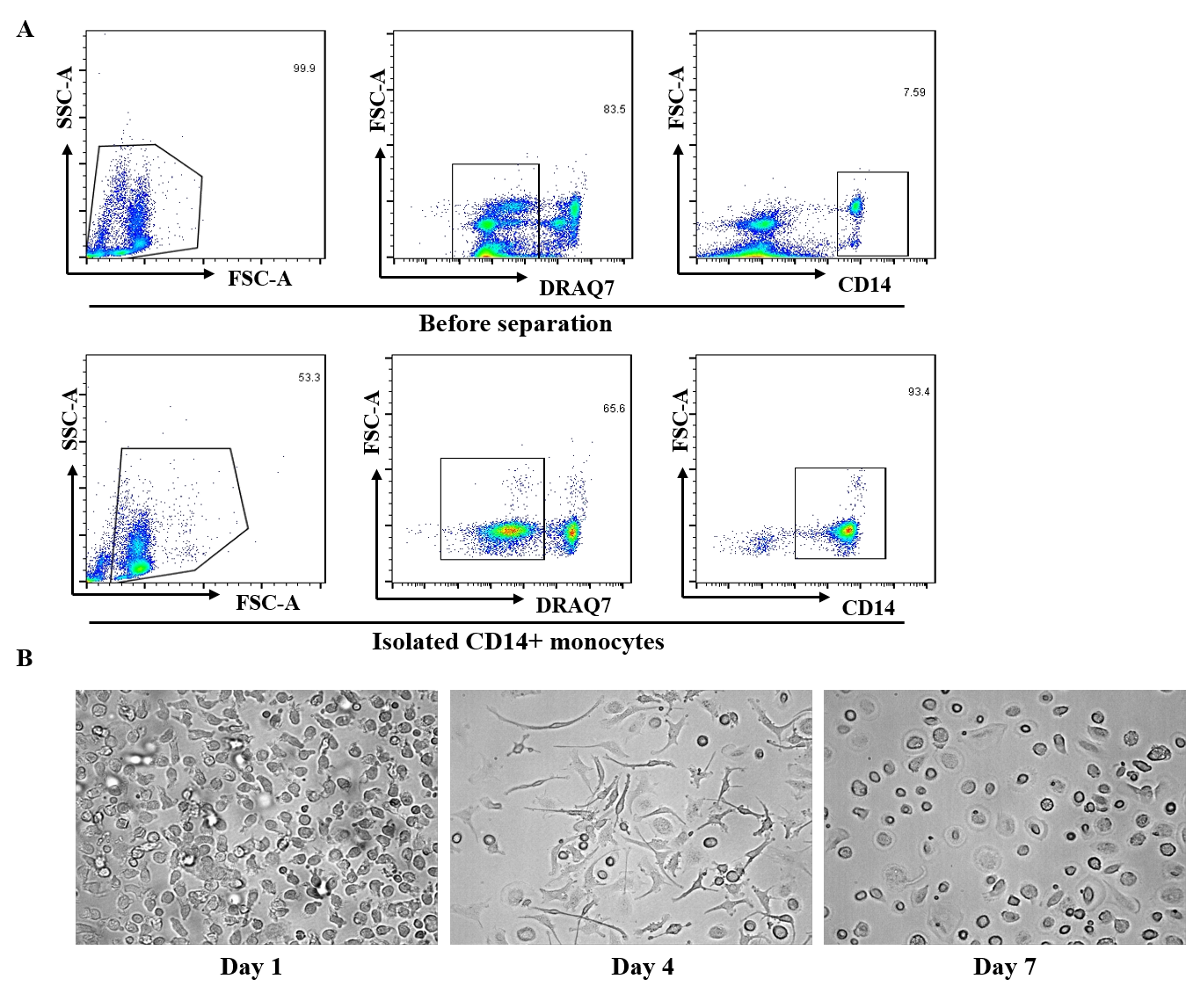

Supplement: Supplementary file 2 — Figure S1 [file 41419_2020_3283_MOESM2_ESM.tif]

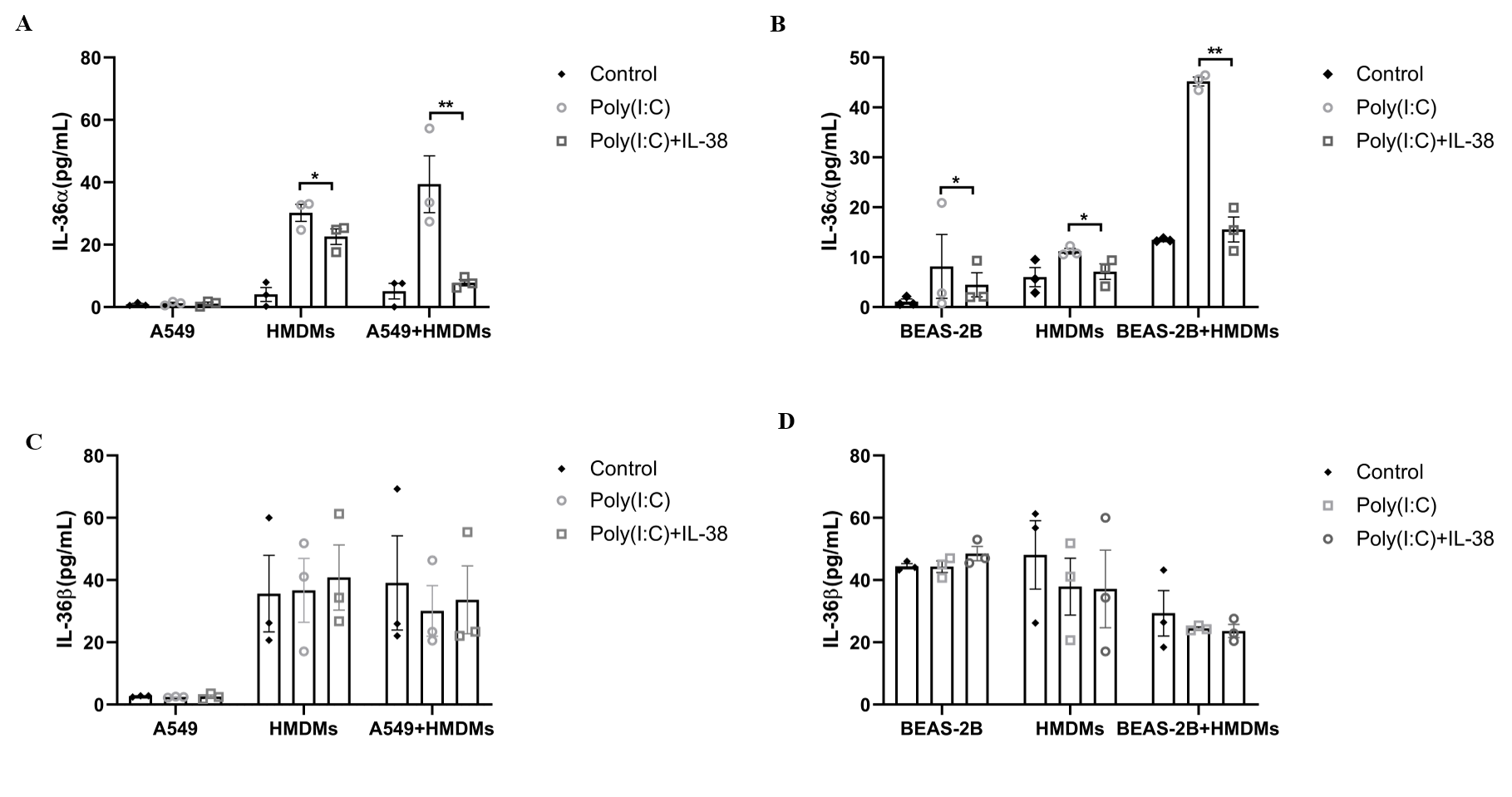

Supplement: Supplementary file 3 — Figure S2 [file 41419_2020_3283_MOESM3_ESM.tif]

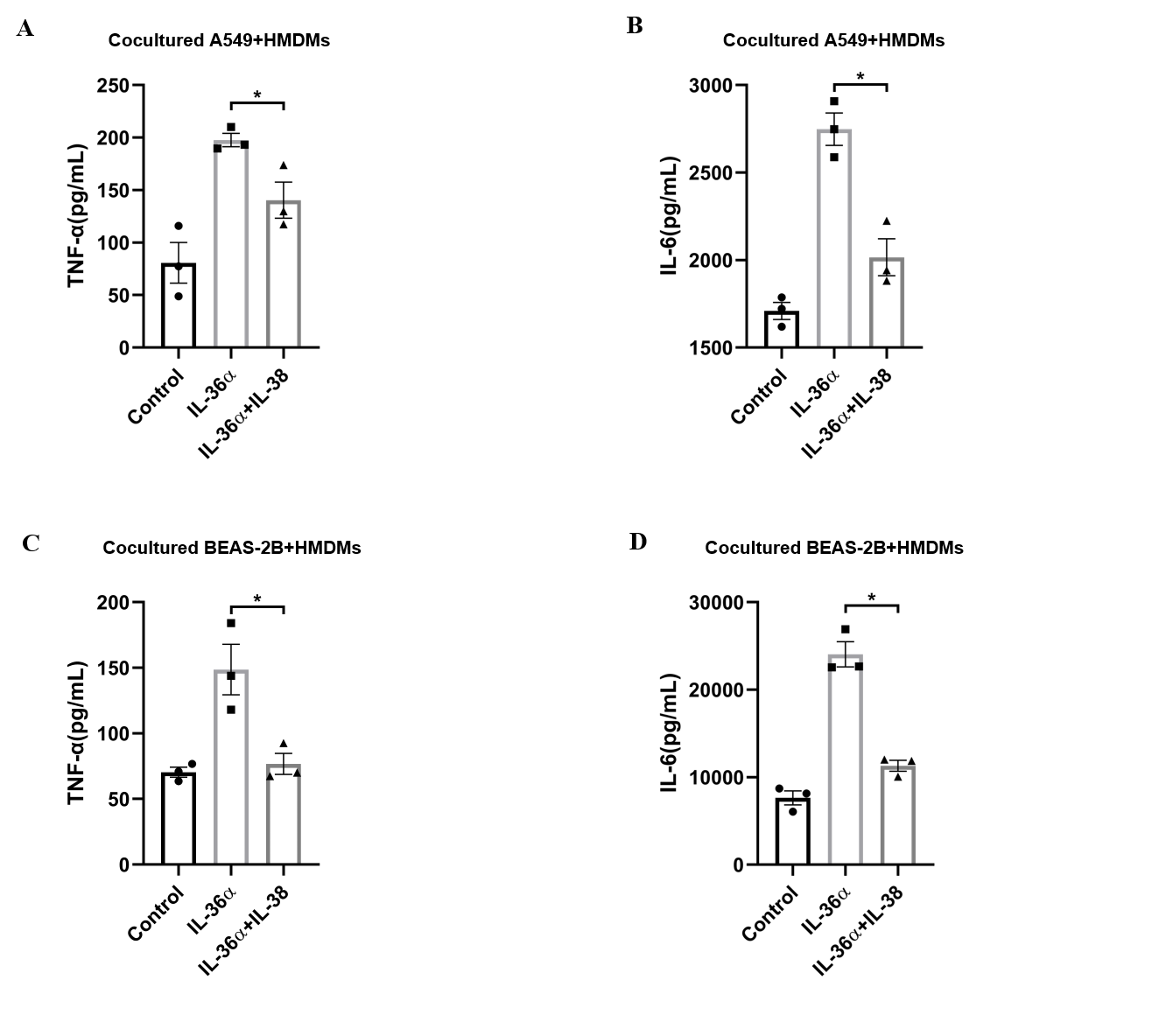

Supplement: Supplementary file 4 — Figure S3 [file 41419_2020_3283_MOESM4_ESM.tif]

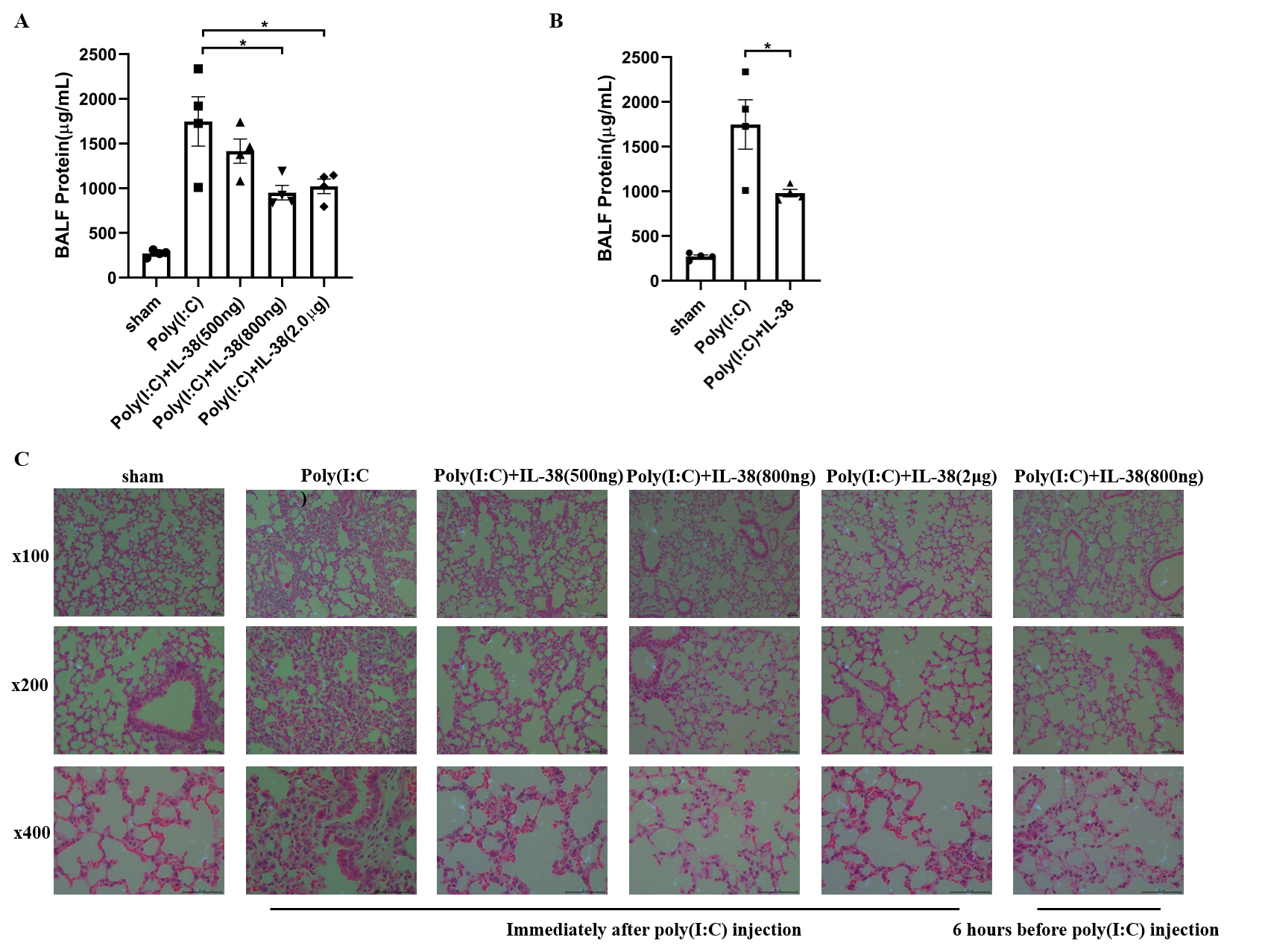

Supplement: Supplementary file 5 — Figure S4 [file 41419_2020_3283_MOESM5_ESM.tif]

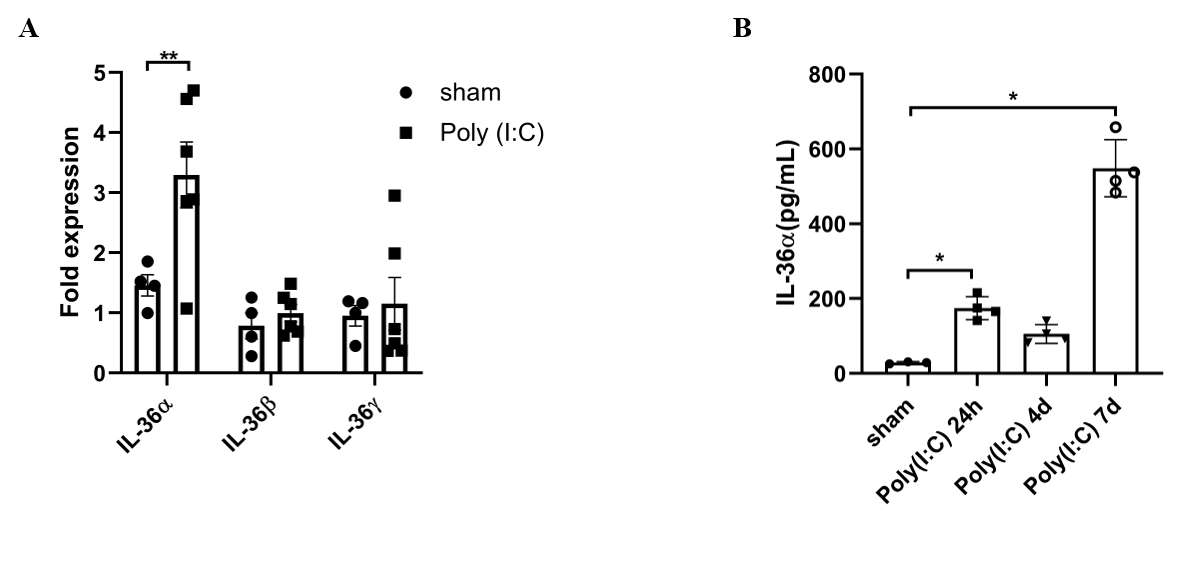

Supplement: Supplementary file 6 — Figure S5 [file 41419_2020_3283_MOESM6_ESM.tif]

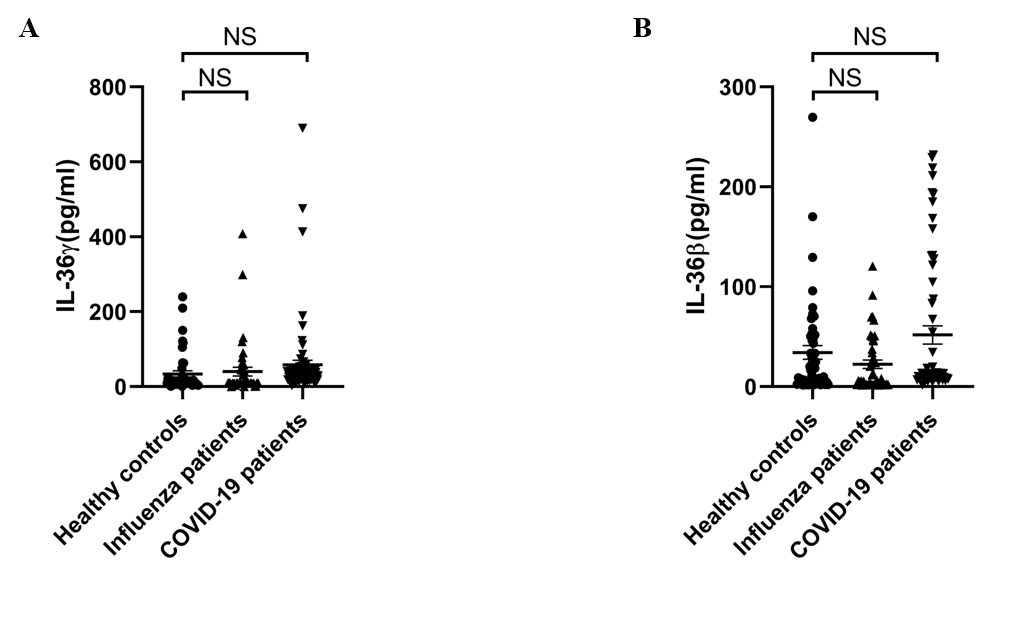

Supplement: Supplementary file 7 — Figure S6 [file 41419_2020_3283_MOESM7_ESM.tif]
