# Supplementary material for: Interleukin-38 ameliorates poly(I:C) induced lung inflammation: therapeutic implications in respiratory viral infections
Source: Cell Death Dis. 2021 Jan 7;12(1):53. doi: 10.1038/s41419-020-03283-2 (PMC7790341; doi:10.1038/s41419-020-03283-2)
Supplement: Supplementary file 8 — Supplemental Table 1 [file 41419_2020_3283_MOESM8_ESM.docx]

**Supplemental Table 1. Sequences of primers used for RT-PCR in the study**

| Genes | Primer sequences (5’-3’) (Forward) | Primer sequences (5’-3’) (Reverse) |
| --- | --- | --- |
| Human IL-38 | CCCCATGGCAAGATACTAC | CCTCTTCTGTCTCCACACAT |
| Human NOX1 | CTGTTGCCTAGAAGGGCTCC | ACAGGCCAATGTTGACCCAA |
| Human FCAMR | TGACTTGAGGTGAGTGGCAG | CCTCTCCTCACCACTTCCTTTT |
| Human COL1A2 | CAGCCGGAGATAGAGGACCA | TGAGCAGCAAAGTTCCCACC |
| Human CTSK | TGGGCTTTTAGCTCTGTGGG | ACATACAACTCTCTTCCTGTCCC |
| Human ADORA1 | GTACTCGCAGCACCACATGA | GGTCACCACCATCTTGTACCTTT |
| Human SPP1 | TCTCCTAGCCCCACAGAATG | TGGTCATGGCTTTCGTTGGA |
| Human RAD51 | GACCGAGCCCTAAGGAGAGT | TGCATTGCCATTACTCGGTC |
| Human MAPK15 | GTAGTGGACCCTCGCATTGTC | CCCGGAATGTTCTCTGGGCAT |
| Human TRIM34 | TTCTGTTGCTCTGAAGCCATCC | TTCTGTCAACAGCTCCAGGC |
| Human IL1R2 | TCCAGAAGCACATGATTGGT | TCAGGCAAGACTTTTATTGGGAG |
| Human NDN | CTGGAAGAAGCACTCCACCT | GCATCTTGGTGATTTCGCGG |
| Human NOTCH4 | CCAACCCTGCGATAATGCGAG | AGTCATCCGTTGAGACCCTGC |
| Human GAPDH | CTCTGCTCCTCCTGTTCGAC | ACGACCAAATCCGTTGACTC |
| Mouse IL-38 | TCAAGGATGCACATCAAAAGGC | TCCCTCCTTCAACGACGGA |
| Mouse GAPDH | GTGCTGAGTATGTCGTGGAG | TAGTGACGGTGAGTCTTCTG |
