# Supplementary material for: Interleukin-38 ameliorates poly(I:C) induced lung inflammation: therapeutic implications in respiratory viral infections
Source: Cell Death Dis. 2021 Jan 7;12(1):53. doi: 10.1038/s41419-020-03283-2 (PMC7790341; doi:10.1038/s41419-020-03283-2)
Supplement: Supplementary file 9 — Supplemental Table 2 [file 41419_2020_3283_MOESM9_ESM.docx]

**Supplemental Table 2. Fold change for the most significantly changed genes upon IL-38 treatment**

| Cocultured A549 with HMDMs | Gene_name | Gene_ID | N_1 | P_1 | PC_1 | C_1 |
| --- | --- | --- | --- | --- | --- | --- |
| Up-regulated: | IL1R2 | ENSG00000115590 | 0 | -2.03 | -0.37 | -2.69 |
|  | CLSTN2 | ENSG00000158258 | 0 | -8.5 | -5.05 | -4.25 |
|  | MME | ENSG00000196549 | 0 | -7.92 | -4.72 | -4.58 |
|  | PCDH18 | ENSG00000189184 | 0 | -6.39 | -4.71 | -4.92 |
|  | NOTCH4 | ENSG00000204301 | 0 | -5.13 | -3.2 | -3.73 |
|  | SLC22A1 | ENSG00000175003 | 0 | -3.87 | -1.52 | -1.81 |
|  | CLDN4 | ENSG00000189143 | 0 | -5.44 | -3.2 | -3.68 |
|  | MTRNR2L8 | ENSG00000255823 | 0 | -3.45 | -1.15 | -1.57 |
|  | PRR4 | ENSG00000111215 | 0 | -3.46 | -0.99 | -1.99 |
|  | NDN | ENSG00000182636 | 0 | -5.61 | -1.72 | -2.55 |
|  | CLDN9 | ENSG00000213937 | 0 | -5.69 | -2.22 | -5.81 |
|  | CCL15 | ENSG00000275718 | 0 | -4.3 | -1.71 | -5.41 |
|  | IGKV2-24 | ENSG00000241294 | 0 | -1.71 | 1.05 | -0.41 |
|  | IGKV1-27 | ENSG00000244575 | 0 | -0.68 | 1.42 | -1.8 |
|  | IGLV4-69 | ENSG00000211637 | 0 | -3.4 | 0.07 | -0.93 |
|  | IGLV2-18 | ENSG00000211664 | 0 | -2.24 | -0.33 | -2.33 |
|  | IGHV4-59 | ENSG00000224373 | 0 | -0.64 | 1.2 | -0.44 |
| Cocultured A549 with HMDMs | Gene_name | Gene_ID | N_1 | P_1 | PC_1 | C_1 |
| Down-regulated: | SNCA | ENSG00000145335 | 0 | -3.2 | -6.78 | -5.78 |
|  | CAMK2B | ENSG00000058404 | 0 | -3.68 | -6.22 | -3.99 |
|  | CLTRN | ENSG00000147003 | 0 | -1.76 | -4.19 | -3.78 |
|  | MAPK15 | ENSG00000181085 | 0 | -3.1 | -4.14 | -4.38 |
|  | TRIM34 | ENSG00000258659 | 0 | -2.38 | -5.2 | -4.2 |
|  | KCNJ8 | ENSG00000121361 | 0 | -1.16 | -3.08 | -2.28 |
| Cocultured BEAS-2B with HMDMs | Gene_name | Gene_ID | N_2 | P_2 | PC_2 | C_2 |
| Up-regulated: | CTSK | ENSG00000143387 | 0 | 1.26 | 2.7 | 0.33 |
|  | ADORA1 | ENSG00000163485 | 0 | -6.86 | -2.48 | -1.84 |
|  | CHIT1 | ENSG00000133063 | 0 | -3.49 | -18 | -6.1 |
|  | HAAO | ENSG00000162882 | 0 | -3.23 | -1.18 | -4.59 |
|  | PPBP | ENSG00000163736 | 0 | 3.97 | 5.5 | 1.34 |
|  | SPP1 | ENSG00000118785 | 0 | 4.54 | 6.58 | 2.36 |
|  | HIST1H4D | ENSG00000277157 | 0 | -0.63 | -0.25 | -0.47 |
|  | CLEC5A | ENSG00000258227 | 0 | -0.77 | 0.79 | -1.19 |
|  | LPL | ENSG00000175445 | 0 | -0.85 | 1.36 | -1.28 |
|  | GAL | ENSG00000069482 | 0 | 1.83 | 3.55 | 0.98 |
|  | GPR84 | ENSG00000139572 | 0 | -1.73 | 0.04 | -1.95 |
|  | RAD51 | ENSG00000051180 | 0 | -3.84 | -2.88 | -4.34 |
|  | ATP6V0C | ENSG00000185883 | 0 | -5.37 | -1.51 | -3.73 |
|  | MT1A | ENSG00000205362 | 0 | -2.45 | 0.51 | -1.81 |
| Cocultured BEAS-2B with HMDMs | Gene_name | Gene_ID | N_2 | P_2 | PC_2 | C_2 |
| Down-regulated: | HBB | ENSG00000244734 | 0 | 3.19 | -1.8 | -2.53 |
|  | COL1A2 | ENSG00000164692 | 0 | -1.86 | -7.22 | -6.63 |
|  | COL3A1 | ENSG00000168542 | 0 | -1.8 | -7.5 | -5.79 |
|  | CA1 | ENSG00000133742 | 0 | -1.39 | -6.29 | -4.23 |
|  | HBA2 | ENSG00000188536 | 0 | 1.93 | -2.46 | -1.56 |
|  | FCAMR | ENSG00000162897 | 0 | -3.8 | -4.32 | -4.64 |
|  | IL3 | ENSG00000164399 | 0 | -0.4 | -2.81 | -2.8 |
|  | SCUBE3 | ENSG00000146197 | 0 | -2.5 | -4.81 | -3.44 |
|  | SFRP4 | ENSG00000106483 | 0 | -3.83 | -5.26 | -7.22 |
|  | MAOB | ENSG00000069535 | 0 | -2 | -4.68 | -6.26 |
|  | NOX1 | ENSG00000007952 | 0 | -2.8 | -3.84 | -5.06 |
|  | SPON1 | ENSG00000262655 | 0 | -2.68 | -5.09 | -5.08 |
|  | LUM | ENSG00000139329 | 0 | -2.68 | -6.39 | -7.22 |
|  | DCN | ENSG00000011465 | 0 | -4.56 | -7.23 | -7.98 |
|  | SLC4A1 | ENSG00000004939 | 0 | -2.75 | -5.52 | -7.09 |
|  | C17orf99 | ENSG00000187997 | 0 | -3.08 | -6.33 | -6.2 |
|  | IGKV1-17 | ENSG00000240382 | 0 | -0.38 | -4.96 | -5.37 |
|  | IGLV7-43 | ENSG00000211652 | 0 | 0.9 | -2.36 | -2.35 |
|  | IGLV3-1 | ENSG00000211673 | 0 | -1.38 | -1.74 | -2.32 |
|  | IGHV3-49 | ENSG00000211965 | 0 | -0.43 | -2.42 | -3.84 |
|  | IGLV3-9 | ENSG00000211670 | 0 | -0.8 | -2.24 | -2.74 |

**Note:** PC, poly(I:C) plus IL-38-treated group; P, poly(I:C)-treated group; N, unstimulated group; and C, IL-38-treated group.
