# Supplementary material for: Interleukin-38 ameliorates poly(I:C) induced lung inflammation: therapeutic implications in respiratory viral infections
Source: Cell Death Dis. 2021 Jan 7;12(1):53. doi: 10.1038/s41419-020-03283-2 (PMC7790341; doi:10.1038/s41419-020-03283-2)
Supplement: Supplementary file 10 — Supplemental Table 3 [file 41419_2020_3283_MOESM10_ESM.docx]

**Supplemental Table 3. The detailed severity scores for different criteria of evaluating poly(I:C)-induced lung injury using a standardized lung injury scoring system**

| Parameter | Poly(I:C) 24h (n=5) | Poly(I:C)+IL-38 24h (n=5) | Poly(I:C) 4d (n=5) | Poly(I:C)+IL-38 4d (n=5) | Poly(I:C) 7d (n=5) | Poly(I:C)+IL-38 7d (n=5) |
| --- | --- | --- | --- | --- | --- | --- |
| A. Neutrophils in the alveolar space | 1  1  0  1  1 | 1  0  1  0  1 | 2  2  1  1  1 | 2  0  0  0  2 | 2  1  1  2  2 | 0  0  1  1  1 |
| B. Neutrophils in the interstitial space | 2  1  2  1  1 | 1  1  1  2  1 | 2  2  1  2  2 | 0  2  2  2  0 | 2  2  2  2  1 | 2  2  1  1  2 |
| C. Hyaline membranes | 1  2  2  1  2 | 0  1  0  1  1 | 0  0  1  1  1 | 1  2  2  2  1 | 1  2  1  0  1 | 2  2  1  1  1 |
| D. Proteinaceous debris filling the airspaces | 1  2  2  2  0 | 0  1  0  1  1 | 0  0  2  2  2 | 1  2  2  2  2 | 0  2  2  0  1 | 2  2  1  1  1 |
| E. Alveolar septal thickening | 0  2  2  0  1 | 2  1  2  0  0 | 1  2  0  2  0 | 0  2  1  1  2 | 2  1  0  1  0 | 1  2  2  2  0 |
| Total Score | 0.62  0.66  0.6  0.55  0.5 | 0.38  0.3  0.38  0.42  0.48 | 0.7  0.72  0.55  0.73  0.69 | 0.54  0.6  0.58  0.58  0.65 | 0.79  0.78  0.69  0.7  0.68 | 0.58  0.6  0.52  0.52  0.62 |

Note: total score=[(20×A) / (14×B) / (7×C) / (7×D) / (2×E)] / (number of fields / 100)
