# Supplementary material for: Interleukin-38 ameliorates poly(I:C) induced lung inflammation: therapeutic implications in respiratory viral infections
Source: Cell Death Dis. 2021 Jan 7;12(1):53. doi: 10.1038/s41419-020-03283-2 (PMC7790341; doi:10.1038/s41419-020-03283-2)
Supplement: Supplementary file 11 — Supplemental Table 4 [file 41419_2020_3283_MOESM11_ESM.docx]

**Supplemental Table 4. Demographics and characteristics of patients with influenza and healthy controls**

| Characteristics | Influenza patients (N=50) | Healthy controls  (N=59) |
| --- | --- | --- |
| Sex, male | 31 | 37 |
| Age, year | 57±20 | 51±18 |
| Influenza viruses |  |  |
| A(HIN1) pdm09 | 8 | N/A |
| A(H3N2) | 35 | N/A |
| B | 7 | N/A |
| SARS-CoV-2 | N/A | N/A |
| Complications | 36 | N/A |
| Anti-virus therapy | 50 | N/A |
| Co-morbidities | 23 | N/A |
| Respiratory care(oxygen) | 16 | N/A |
| Duration of hospitalization | 4.5(3-6) | N/A |
| Death | 0 | N/A |

Note:

Co-morbidities: Coronary heart disease, hypertension, cerebrovascular, Stroke, neoplastic, chronic lung, liver and renal diseases, diabetes mellitus, autoimmune disorders, allergic diseases.

Complications: clinico-radiographic pneumonia, bronchitis, acute exacerbation of chronic pulmonary diseases; acute cardiovascular/cerebrovascular events, renal and metabolic derangements.
