# Supplementary material for: Interleukin-38 ameliorates poly(I:C) induced lung inflammation: therapeutic implications in respiratory viral infections
Source: Cell Death Dis. 2021 Jan 7;12(1):53. doi: 10.1038/s41419-020-03283-2 (PMC7790341; doi:10.1038/s41419-020-03283-2)
Supplement: Supplementary file 12 — Supplemental Table 5 [file 41419_2020_3283_MOESM12_ESM.docx]

**Supplemental Table 5. Demographics and characteristics of patients infected with SARS-CoV-2 infections**

| Characteristics | COVID-19 patients  (N=85) | Mild  patients  (N=59) | Severe patients  (N=26) |
| --- | --- | --- | --- |
| Sex, male | 41 | 29 | 12 |
| Age, year | 38±17 | 15±16 | 39±18 |
| Complications | 6 | 0 | 6 |
| Anti-virus therapy | 43 | 17 | 26 |
| Co-morbidities | 20 | 4 | 16 |
| Respiratory care(oxygen) | 5 | 0 | 5 |
| Duration of hospitalization | 9.1(2-22) | 7(2-14) | 12(7-22) |
| Death | 0 | 0 | 0 |

Note:

Co-morbidities: Coronary heart disease, hypertension, cerebrovascular, Stroke, neoplastic, chronic lung, liver and renal diseases, diabetes mellitus, autoimmune disorders, allergic diseases.

Complications: clinico-radiographic pneumonia, bronchitis, acute exacerbation of chronic pulmonary diseases; acute cardiovascular/cerebrovascular events, renal and metabolic derangements.
